# Medium-Range Structural Order in Amorphous Arsenic

**DOI:** 10.1021/jacs.5c18688

**Published:** 2026-02-26

**Authors:** Yuanbin Liu, Yuxing Zhou, Richard Ademuwagun, Luc Walterbos, Janine George, Stephen R. Elliott, Volker L. Deringer

**Affiliations:** † Inorganic Chemistry Laboratory, Department of Chemistry, 6396 University of Oxford, Oxford OX1 3QR, U.K.; ‡ Materials Chemistry Department, Federal Institute for Materials Research and Testing (BAM), Unter den Eichen 87, Berlin 12205, Germany; § Institute of Condensed Matter Theory and Solid-State Optics, Friedrich Schiller University Jena, Max-Wien-Platz 1, Jena 07743, Germany; ∥ Physical and Theoretical Chemistry Laboratory, Department of Chemistry, University of Oxford, Oxford OX1 3QZ, U.K.

## Abstract

Medium-range order (MRO) is a key structural feature of amorphous materials, but its origin and nature remain elusive. Here, we reveal the MRO in amorphous arsenic (*a*-As) using advanced atomistic simulations, based on machine-learned potentials derived using automated workflows. Our simulations accurately reproduce the experimental structure factor of *a*-As, especially the first sharp diffraction peak (FSDP), which is a signature of MRO. We compare and contrast the structure of *a*-As with that of its lighter homologue, red amorphous phosphorus (*a*-P): we find that *a*-As has a more uniform dihedral-angle distribution, and so we confirm that its structure can be thought of as a 3-fold coordinated continuous random network in first approximation, in contrast to the more molecular-cluster-like structure of *a*-P. The pressure-dependent structural behaviors of *a*-As and *a*-P differ as well, and the origin of the FSDP is closely correlated with the size and spatial distribution of voids in the amorphous networks. Our work provides fundamental insights into MRO in an amorphous elemental system, and more widely it illustrates the usefulness of automation for machine-learning-driven atomistic simulations.

## Graphical abstract

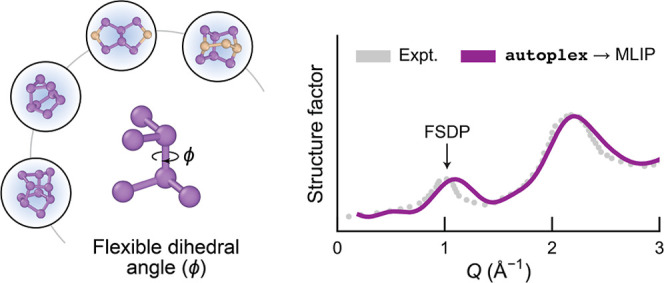

## Introduction

The central structural feature of amorphous materials, beyond the short-range order of nearest-neighbor coordination shells, is the medium-range or intermediate-range order (MRO/IRO) at distances of 5–20 Å.^1^ For many years, studies of MRO have advanced our fundamental understanding of amorphous solids, and now such studies could help to “design” amorphous functional materials^2^ based on the correlation of MRO with macroscopic properties.^3,4^ For example, enhanced MRO in vapor-deposited GeO_2_ glass, identified through Raman spectra, was shown to be associated with reduced room-temperature internal friction.^5^ In addition, an increase of MRO has been linked to increased thermal conductivity in *a*-Si,^6^ *a*-Ga_2_O_3_,^7^ and *a*-C.^8^ Experimental techniques, such as X-ray or neutron scattering, have been widely used to probe MRO for different systems. In particular, the first sharp diffraction peak (FSDP) in the structure factor, *S*(*Q*), has long been regarded as a signature of MRO.^9–11^ However, extracting and interpreting structural information about MRO from experimental scattering data is nontrivial, which is further complicated by its sensitivity to pressure^12,13^ and compositional variations.^14^

An interesting fundamental question is what similarities or differences exist in the MRO of elemental glasses within the same group of the Periodic Table. In the present work, we will address this question for two group-15 elements, phosphorus (P) and arsenic (As). The crystalline allotropes are now well understood, and P is particularly rich in structures: exhibiting puckered layers in black P, complex tubular structures in violet P, and tetrahedral P_4_ molecules in white P. The heavier homologue, As, also adopts diverse structures, from gray As, which is isostructural with high-pressure rhombohedral P, to yellow As comprising As_4_ molecules. In terms of the disordered state, amorphous phosphorus (*a*-P) displays pronounced MRO, characterized by clusters formed primarily of five-membered rings.^13,15,16^ In contrast, the MRO of amorphous arsenic (*a*-As) and its structural relationships with the crystalline allotropes remain to be explored.

Insights into the structure of amorphous materials are increasingly obtained from molecular-dynamics (MD) simulations which can directly probe MRO at the atomic scale, complementing experiments. By topological and geometrical analyses of MD trajectories, such as primitive ring statistics,^17^ Voronoi tessellation,^18^ or persistent homology,^19^ one can identify structural motifs contributing to MRO. However, the reliability of this approach strongly depends on the accuracy of the force predictions used to drive the simulations, and modeling at the level of density-functional theory (DFT) has been limited to rather small simulation-cell sizes.^20–23^ Recent, rapid progress in machine-learned interatomic potentials (MLIPs)^24–26^ has unlocked the ability to simulate amorphous materials at large length-scales and for long simulation times. While developing MLIPs for amorphous materials has traditionally required extensive domain expertise and manual data curation,^27,28^ the emergence of automated workflows is now poised to substantially accelerate the construction of MLIPs.^29–32^ This means that simulations that would previously have required the careful construction of a hand-crafted MLIP model can now be carried out much more quickly than before.

Here, we describe an efficient approach for modeling and understanding MRO in the amorphous state, demonstrated for the “textbook” case of two seemingly chemically similar elements. Specifically, we show how random structure searching (RSS), implemented within automated workflows^32–34^ and refined by a few iterations of MD, yields an MLIP model for *a*-As with only very moderate computational effort. We first validate our MLIP by quantitatively comparing the computed structure factor of *a*-As—and especially the FSDP—with experimental data. We then use ML-driven MD simulations to uncover the MRO in *a*-As and to draw a comparison to recent studies of *a*-P.^15,16^ Specifically, by examining the dihedral-angle distributions in both *a*-As and *a*-P, we identify geometric motifs responsible for differences in the MRO of both elements. Beyond the specific insight into *a*-As, our work contributes to a deeper understanding of MRO in amorphous materials and its link to the experimentally observable FSDP more generally, and shows how such an understanding can be obtained with the help of automated atomistic machine learning.

## Results

### Machine-Learned Potentials for Arsenic

The starting point for our studies was to use the Gaussian Approximation Potential (GAP) framework^25,35,36^ to iteratively explore and sample the potential-energy surface of elemental As, using small cells of up to 24 atoms (Figure 1A). Previous studies have shown that such GAP-driven random structure searching (GAP-RSS) can efficiently capture diverse atomic environments and yield robust potentials at low computational cost.^32,33,37^ Here, the RSS processes were carried out automatically using the autoplex framework that we have developed recently.^32^ After accumulating a data set of 1500 RSS-generated configurations (see Methods for details of the diversity analysis), we refitted the potential-energy surface using the MACE architecture.^38^ The rationale for doing so is that GAP is data-efficient, and thus particularly suitable for initial RSS, whereas MACE achieves higher accuracy once sufficient data are available; a similar staged approach was used previously to build MLIPs for graphene oxide.^39^ We evaluated our initial MACE model for As, trained on the pure RSS data set, through melt-quench MD simulations yielding structural models of *a*-As at 300 K. The *S*(*Q*) data calculated for those models were then compared with two experimental data sets: one reported by Smith et al.,^40^ constructed from combined X-ray^41^ and neutron data, and one reported by Bellissent and Tourand,^42^ obtained from neutron-scattering measurements. Although the comparison shows overall agreement with both sets of experimental data, the simulated FSDP height is underestimated (Figure 1B).

**Figure 1:**
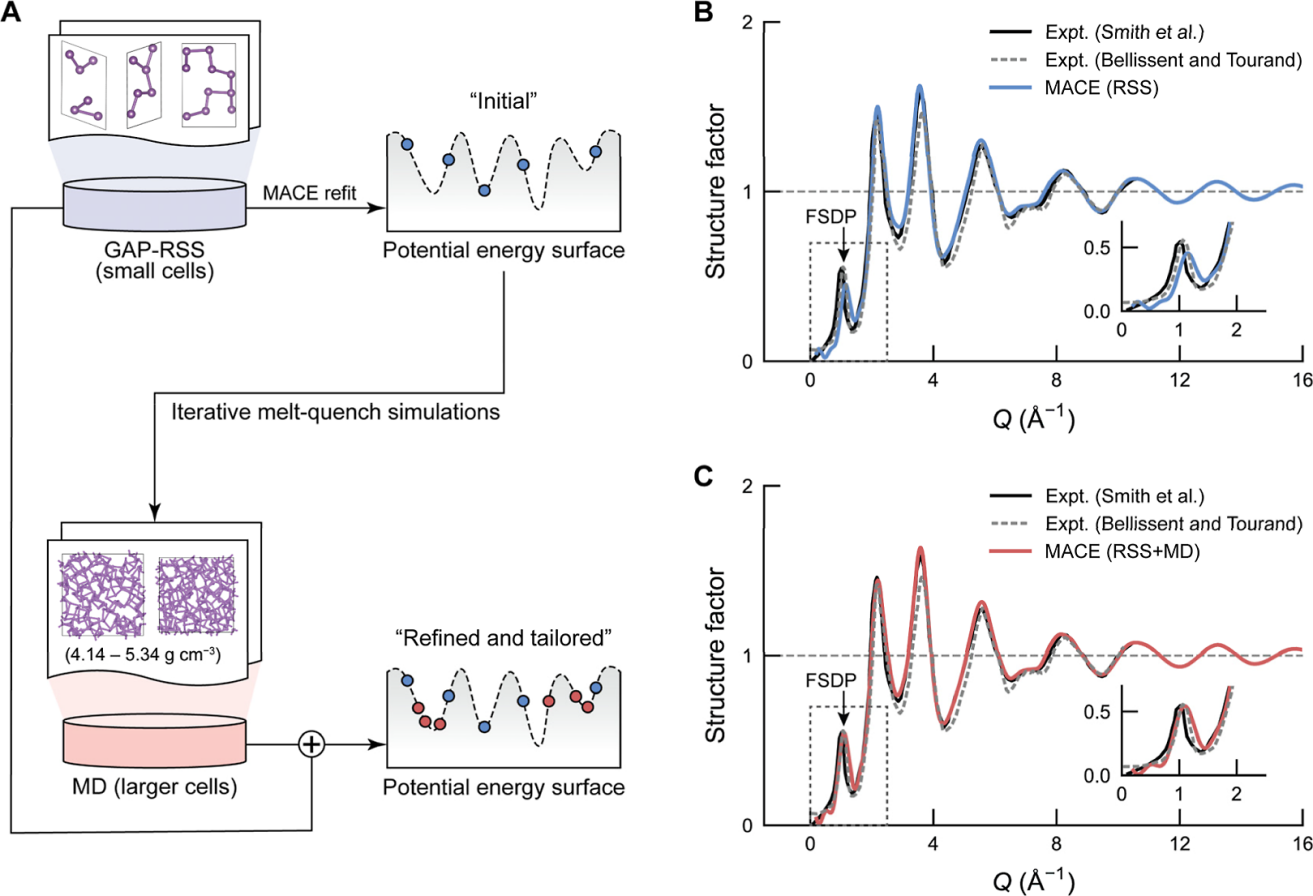
Machine-learning-driven simulations of *a*-As. (A) Schematic workflow for developing MLIP models for As. In the first stage, the autoplex framework was used to perform GAP-RSS on small cells, generating initial training data, to which we then fitted a graph-based MACE model. In the second stage, the model was iteratively refined by MD melt-quench simulations on larger (216-atom) cells across a density range of 4.14–5.34 g cm^–3^. (B,C) Simulated structure factors for structural models of *a*-As, generated using the two MLIP variants, as compared to experimental data digitized from Smith et al.^40^ as well as from Bellissent and Tourand.^42^ Panel B shows results for the initial MACE potential trained on RSS data only, while panel C shows results for the refined potential that also incorporates MD data. The refined potential improves the agreement with experimental data for the FSDP (arrows and insets in both panels).

To enhance the representation of MRO in the training data, we next carried out iterative MD refinements in larger, 216-atom cells, using the RSS-derived MACE model as the starting point. Each iteration involved simulations across 5 distinct densities, from 4.14 to 5.34 g cm^–3^ at intervals of 0.3 g cm^–3^. We selected this range to encompass the experimental density of *a*-As at ambient pressure (4.74 g cm^–3^).^40^ After four iterations, the refined potential yielded a model structure giving excellent agreement with the experimental *S*(*Q*) data, notably reproducing the FSDP height with a relative error below 1% (Figure 1C). We note that the additional MD-based refinement mainly enhanced the intensity of the predicted FSDP, while the rest of the curve is very similar to that predicted by the purely RSS-trained model (Figure S1). In addition, the extra minimum in *S*(*Q*) at very low *Q* from different simulations is a finite-size artifact arising from periodic boundary conditions.

To place our results in methodological context, we compared them to those of a recent atomistic foundation model, MACE-MPA-0,^43^ which was designed for broad applicability across diverse chemical systems. While the zero-shot foundation model (i.e., without fine-tuning) qualitatively captures the main features of the experimental structure factor for *a*-As, it overestimates the height of the FSDP while simultaneously underestimating that of the second peak (Figure S2). This comparison underscores that, while foundation models provide a valuable baseline, achieving high fidelity for amorphous systems still requires a targeted training and refinement strategy. Our present work is concerned with building MLIP models “from scratch”, but we mention in passing that the same methodology could also provide a pathway for fine-tuning existing foundation models, as relevant training data can be generated with high computational efficiency and minimal human intervention. Specifically, the RSS stage in the present work required <15,000 CPU core hours, and the total computational cost for both the RSS and MD stages was <50,000 CPU core hours.

The cost-efficiency of our approach also makes it suited for comparing and benchmarking different exchange–correlation functionals to be used for generating training data (Figure 2). In addition to r^2^SCAN,^44^ we generated separate MLIP models with TPSS^45^ and r^2^SCAN + rVV10,^46^ respectively, using the same protocol otherwise. Note that each individual MLIP was constructed from a fully consistent reference data set, based on a single functional and generated entirely from scratch. The r^2^SCAN + rVV10 approach augments r^2^SCAN with a nonlocal van der Waals correlation term, originally designed to improve the treatment of long-range dispersion interactions.^46^ While this extension benefits layered materials,^47^ we find that, in the present case of *a*-As, it alters medium-range correlations in a way that severely underestimates the intensity of the FSDP (Figure 2). TPSS is closer to the r^2^SCAN predictions, but still overestimates the intensity of the second *S*(*Q*) peak (Figure 2). These results underline how sensitive the simulation of amorphous structures can be to the choice of functional, and our automated framework offers a practical and economic route to address this important challenge. More details on the GAP-RSS procedure, MLIP fitting, and melt-quench protocols are provided in the Methods section.

**Figure 2:**
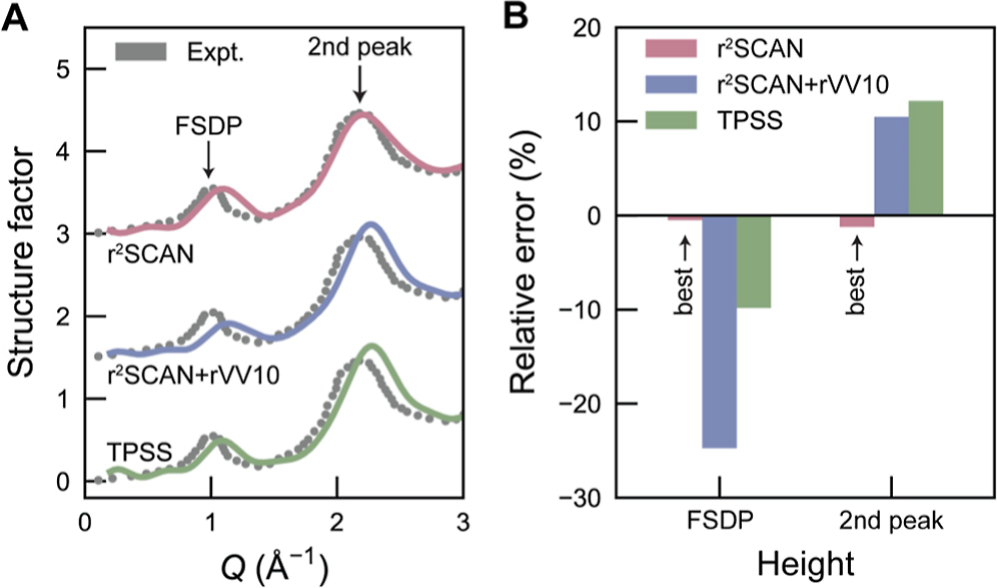
Benchmarking meta-GGA functionals using the structure factor of *a*-As. (A) Comparison of the experimental structure factor^40^ with those obtained from models trained on the r^2^SCAN, r^2^SCAN + rVV10, and TPSS functionals, highlighting the first sharp diffraction peak (FSDP) and the second peak. (B) Relative errors in the FSDP and second-peak heights for the three functionals. Among the tested functionals, r^2^SCAN provides the closest agreement with experiment, TPSS produces intermediate deviations with an overestimation of the second-peak height, and r^2^SCAN + rVV10 markedly underestimates the FSDP while overestimating the second-peak height.

### Medium-Range Structural Order

Our main MLIP model was used to generate 2000-atom models of *a*-As (cell length ≈ 38 Å), and we first probed the short-range structural order, as described by radial distribution functions and bond-angle distributions, at ambient conditions. Figure 3A presents the radial distribution functions, *J*(*r*), for *a*-As. We also include results for an earlier simulation of *a*-P,^15^ in which amorphous structures were obtained using an MLIP^48^ trained on PBE + MBD data,^49–51^ noting that the latter is a different ground-truth level compared to the present work. To carry out a side-by-side comparison and account for the different bond lengths, the *J*(*r*) data for *a*-P and *a*-As were rescaled to the position of the respective first peak. In this, *a*-P exhibits a slightly narrower first peak and a broader subsequent minimum (approaching zero) than *a*-As, indicating a more uniform bond-length distribution and a more well-defined first coordination shell. The second peak in *a*-P is remarkably sharp and intense, concomitant with a narrow bond-angle distribution (Figure 3B); in contrast, there is a less strong second-nearest-neighbor peak in *a*-As, and greater angular disorder. Well-defined third main *J*(*r*) peaks for *a*-P and *a*-As show a clear hallmark of MRO. We also provide a direct comparison between the calculated *J*(*r*) and experimental data for *a*-As, further demonstrating the accuracy of our MLIP (Figure S3).

**Figure 3:**
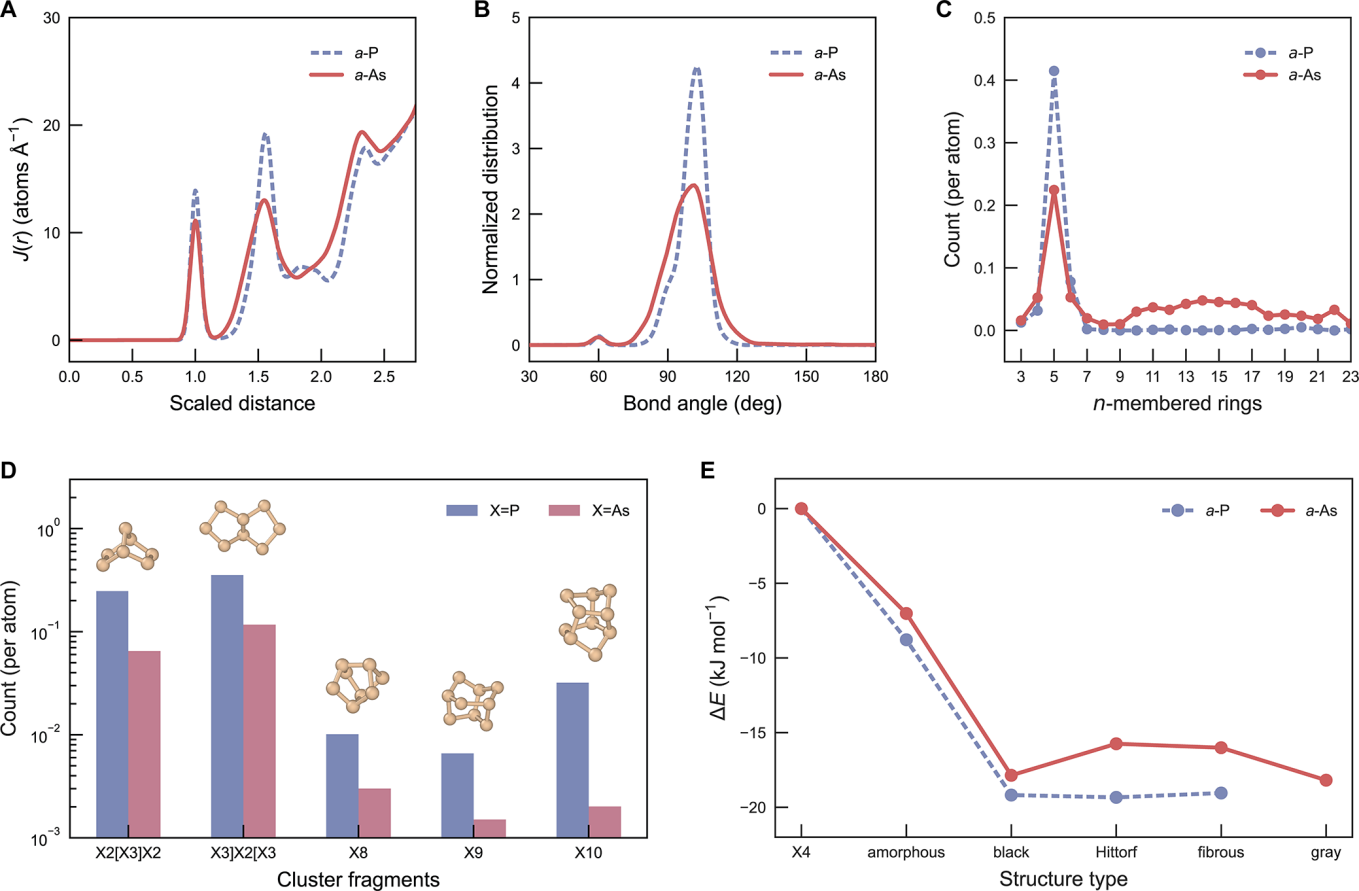
Structural and energetic properties of *a*-As at ambient pressure, compared with *a*-P. (A) Radial distribution functions, *J*(*r*), of *a*-P (dashed purple line; structure from ref 15) and *a*-As (solid red line). The distance, *r*, is scaled by the first peak position in *J*(*r*). (B) Bond-angle distributions for *a*-P and *a*-As. The plot shows the probability-density functions of the total bond-angle distributions calculated for all atoms in the systems. (C) Distribution of primitive rings. (D) Counts of cluster fragments. It can be observed that the proportions of different types of clusters are higher in *a*-P than in *a*-As. (E) The energetics of arsenic and phosphorus allotropes, including the well-known X_4_ molecular phases (yellow for X = As, white for X = P, used as the reference state in both cases), as well as violet (Hittorf-type) and fibrous forms. The hypothetical Hittorf-type and fibrous As structures were derived from the corresponding phosphorus phases^16^ and subsequently relaxed using DFT. Note that the energetics of all As forms were computed at the r^2^SCAN + rVV10 level of theory, whereas the P data were taken from ref 16 (data for structural model **1** are shown for *a*-P), which used the HSE06 + MBD approach^50,51,55^ for the energy calculations.

One important indicator of MRO is the distribution of primitive rings (Figure 3C). Notably, 5-membered rings dominate the network topology of both *a*-P and *a*-As. This can be attributed to the fact that both materials exhibit bond-angle distributions consistent with 5-membered ring formation (Figure S4). However, *a*-P contains a significantly higher proportion of 5-membered rings than *a*-As, consistent with the greater probability density for their requisite bond angles (Figures 3B,C and S4). Interestingly, 5-membered rings are also the primary structural motif in violet (Hittorf’s)^52^ and fibrous (Ruck’s) phosphorus.^53^ Compared to *a*-P, for which the proportion of large rings rapidly diminishes beyond *n* = 6, *a*-As contains a larger proportion of extended-size rings (*n* > 6) (Figure 3C).

Another signature of MRO can be the formation of fragment clusters. Such clusters, composed of 5-membered rings, following Baudler’s rules^54^ were previously reported in *a*-P^15^ and are also observed here in *a*-As (Figure 3D). Typical examples of these clusters range from the motifs X2­[X3]­X2 (two fused 5-membered rings sharing three atoms) and X3]­X2­[X3 (two fused 5-membered rings sharing two atoms) to cage-like X8, X9, and X10 fragments (X = P, As). However, *a*-As exhibits a lower abundance of these clusters (Figure 3D), which can be partly attributed to the limited number of 5-membered rings in *a*-As, which reduces the available building blocks for cluster formation.

Continuing our comparison of both elements, we examined the energetic stability of crystalline and amorphous phases of As. In addition to the gray, black, and yellow allotropes, we also included hypothetical As phases that are isostructural with violet (Hittorf’s) and fibrous P, constructed by elemental substitution and subsequent full structural relaxation. We note that violet P can be synthesized from amorphous red P,^56^ but no similar synthesis pathway starting from *a*-As has been reported to our best knowledge. In a benchmark comparison of DFT functionals for crystalline forms of As (Table S1), we found that r^2^SCAN, due to its lack of long-range (van der Waals) interaction description, fails to correctly optimize the structure of layered gray arsenic. In contrast, the r^2^SCAN + rVV10 functional provides higher accuracy for crystalline structures and was therefore employed here to fully relax all crystalline structures. Given the substantial computational cost, we relaxed the 500-atom *a*-As structures using MLIPs, followed by single-point energy evaluations with r^2^SCAN + rVV10. This approach allows us to directly compare the energies of amorphous and crystalline phases. Our results show that hypothetical, Hittorf-type and fibrous phases of arsenic are energetically less stable than the known gray and black allotropes (albeit they are more favorable than *a*-As). The gray phase exhibits the highest energetic stability, consistent with previous literature.^57^ White P and yellow As are both higher in energy than their respective amorphous counterparts. Projected Crystal Orbital Hamilton Population (COHP) analysis^58,59^ using LOBSTER^60^ and additional automated postprocessing with LobsterPy^61^ was carried out to gain additional insight into the electronic structure and chemical-bonding properties of the relevant structures, including *a*-As (Supporting Information Text and Figures S5 and S6).

### Role of Dihedral Angles in MRO

Group-15 elements typically adopt 3-fold-coordinated p-bonding environments, in accord with their s^2^p^3^ valence configurations. In the crystalline phases of As, atoms are all 3-fold coordinated, whereas in their amorphous counterpart, occasional overcoordinated sites (4- and 5-fold) may occur as structural defects, and the population of these tends to increase under pressure (Figure S7).

The predominant 3-fold coordination in both crystalline and amorphous phases of As and P gives rise to numerous “dumbbell-like” building units, each formed by two connected trigonal units (Figure 4A). We quantified the torsional geometry of dumbbell units using a defined dihedral angle, ϕ (Methods). Rhombohedral gray arsenic exhibits a single dihedral angle of 180°, whereas the orthorhombic black modification is characterized by three discrete ϕ values of ±78.7° as well as 180°, consistent with the formation of puckered zigzag chains. In contrast, *a*-As displays a higher structural (torsional) flexibility, with dumbbell dihedral angles spanning a broader, more uniform distribution (Figure 4B). Interestingly, while *a*-P also shows a higher torsional flexibility than its crystalline counterparts, its dumbbells mostly feature dihedral angles of |ϕ| < 90°. To explain the uniformity of the dihedral-angle distribution in *a*-As, we explored their relationship with average bond energies (ICOHPs) in Figure S6B–D. The weak correlation indicates that the bond energy is largely independent of the dihedral angle, resulting in a more uniform angular distribution. In contrast, *a*-P shows a clear dependence of bond energies with dihedral angle, favoring configurations near ±55° (Figure S8).

**Figure 4:**
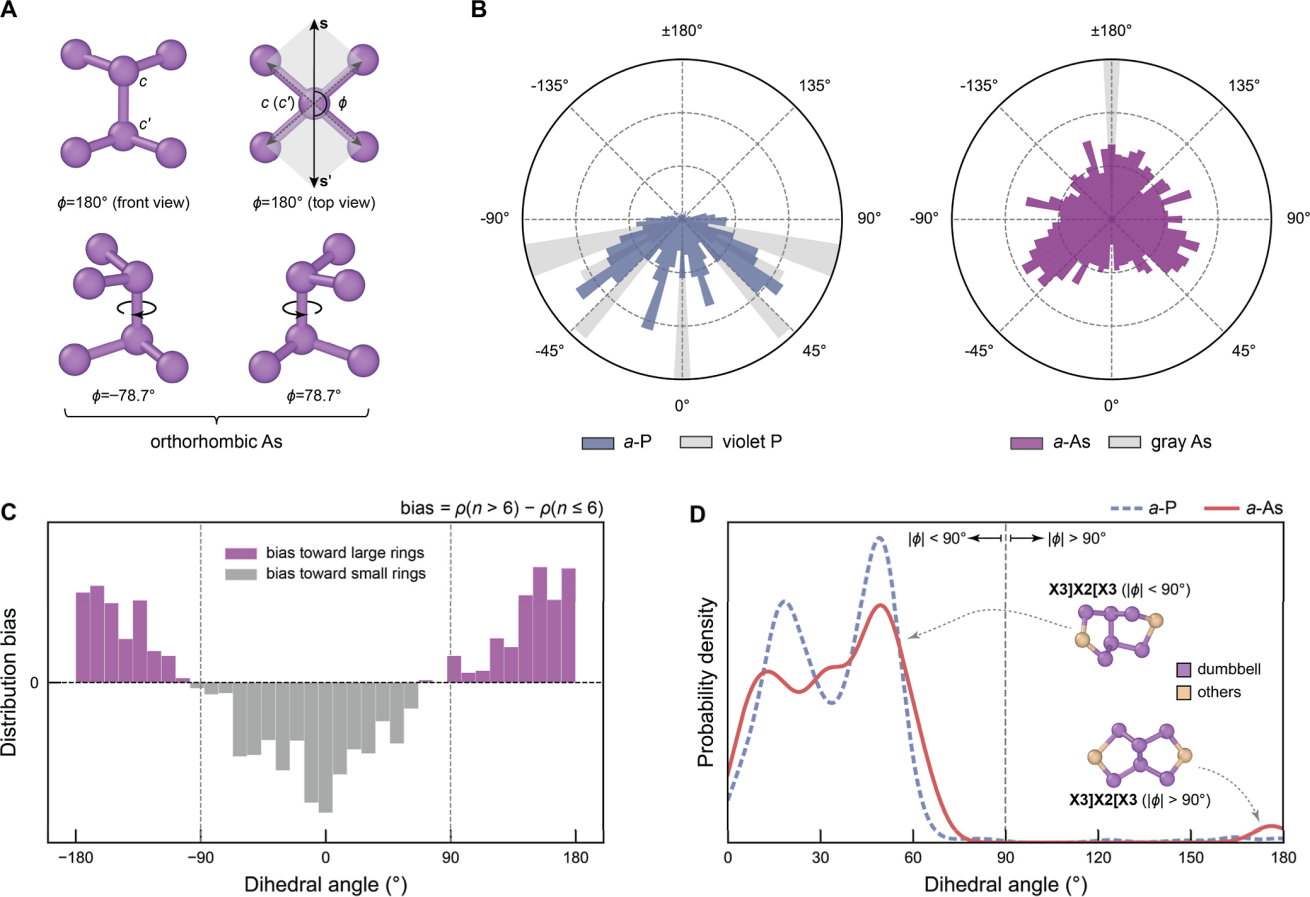
Analysis of dumbbell dihedral angles. (A) Illustration of typical “dumbbell” building units in crystalline orthorhombic (black) arsenic. We use a custom definition for the dihedral angle which is specific to pairs of 3-fold-bonded atoms, as sketched here and detailed in the Methods section. A value of this angle of ϕ = 180° corresponds to an antialigned configuration, with the As atoms bonded to each central As positioned opposite to each other along the *c*–*c*′ axis. (B) Distributions of those dihedral angles in *a*-P (left) and *a*-As (right), together with those for representative crystalline structures. (C) Distribution bias in dihedral angles between large (*n* > 6) and small (*n* ≤ 6) rings in *a*-As. The bias is calculated as the difference between the normalized dihedral-angle distributions of dumbbells in large rings and those in small rings. (D) Distributions of dihedral angles in X3]­X2­[X3 fragment clusters, the most prevalent cluster type, shown as kernel density estimates for *a*-P (dashed purple line) and *a*-As (solid red line).

The formation of extended-size rings (*n* > 6) in *a*-As, as opposed to *a*-P, can be attributed to its broader distribution of dumbbell dihedral angles, which enables greater structural flexibility. This finding was further confirmed by analyzing the dihedral-angle distribution bias, which we define as the difference between normalized dihedral-angle distributions for extended (*n* > 6) and small (*n* ≤ 6) rings, respectively (Figure 4C). This analysis shows that small rings are preferentially formed from dumbbells with |ϕ| < 90°, while larger rings predominantly include dumbbells with |ϕ| > 90° to generate their extended geometry. Since *a*-As contains a higher proportion of large-ϕ dumbbells than *a*-P (Figure 4B), it has a greater propensity to form larger rings.

Regarding the formation of fragment clusters, in addition to the influence of 5-membered rings on the availability of building blocks, the distribution of dumbbell dihedral angles also plays a significant role in determining the difference in cluster populations between *a*-As and *a*-P. Clusters such as X8, X3]­X2­[X3, X9, and X10 (X = P, As) contain varying numbers of dumbbell units (see insets in Figure 3D), and these compact clusters largely require dumbbells with |ϕ| < 90°. The latter is quantitatively illustrated for the dominant cluster type, X3]­X2­[X3, in Figure 4D. This figure shows that both materials predominantly utilize dumbbells with |ϕ| < 90° to form X3]­X2­[X3 clusters. However, the structure of *a*-P appears to be more restricted to these small-ϕ dumbbells (Figure 4B), which could explain the higher prevalence of these fragment clusters as compared to *a*-As (cf. Figure 3D). We emphasize that the above conclusions hold for structures predicted using MLIPs trained with different meta-GGA functionals, including r^2^SCAN, r^2^SCAN + rVV10, and TPSS (Figure S9).

### Evolution of MRO under Pressure

We next explored the evolution of MRO under pressure, using structures obtained from dedicated compression simulations (see Methods), which again revealed distinct behaviors in ring and cluster fragment distributions between *a*-P and *a*-As. The evolution of 5-membered rings under pressure reveals a distinct contrast between the two materials (Figure 5A). In *a*-P, their fraction remains nearly constant up to moderate pressures and then decreases markedly at higher pressures, reflecting the progressive destabilization of the associated bond-angle geometries (Figure S4). Conversely, *a*-As exhibits a continuous *increase* in the number of 5-membered rings, suggesting that compression promotes the formation and stabilization of this structural motif, despite increasing angular distortions (Figure S4).

**Figure 5:**
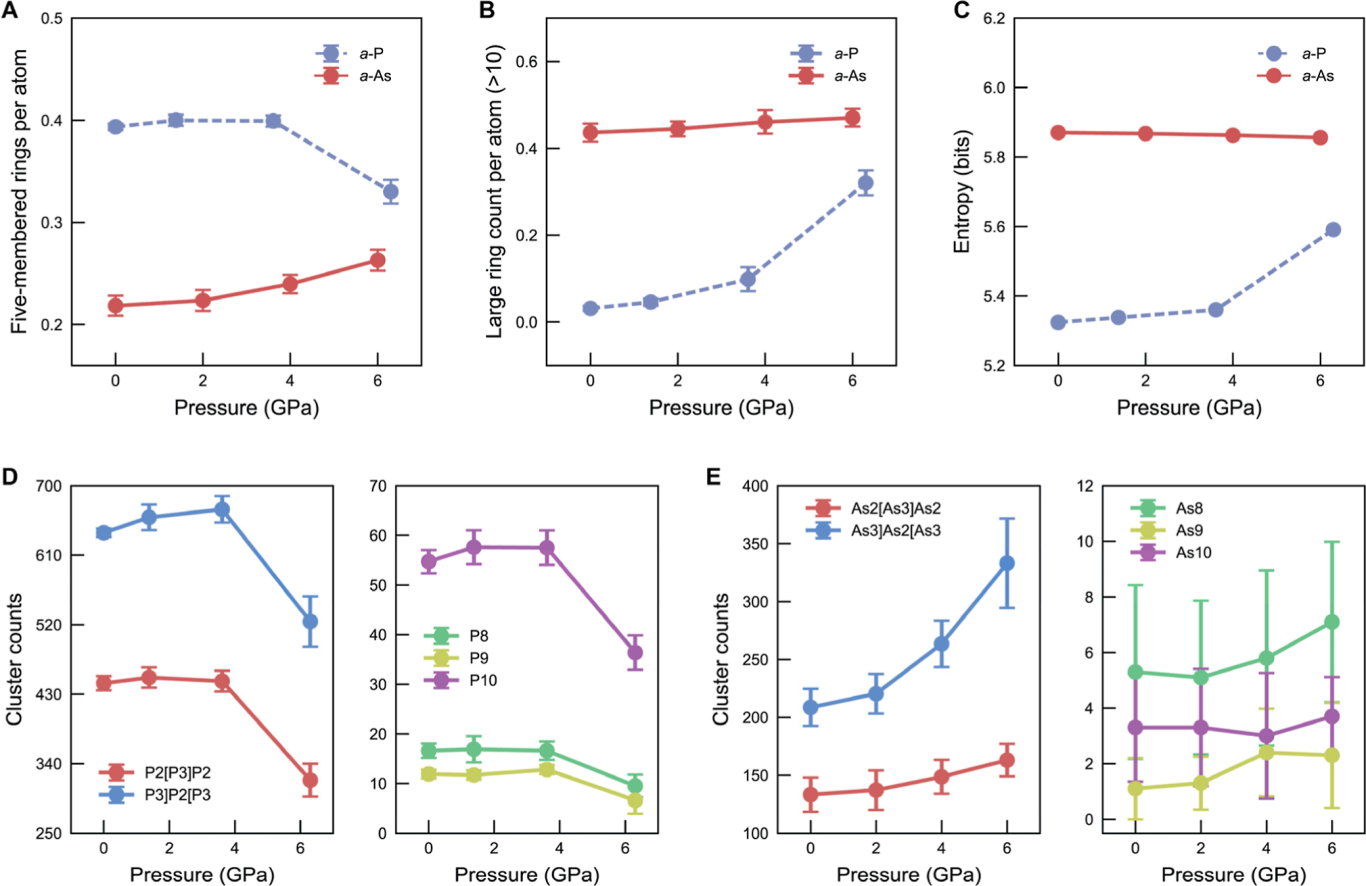
Pressure-dependent structural evolution. (A) Count of five-membered rings under pressure for *a*-P and *a*-As. (B) Large-ring count per atom (*n* > 10) versus pressure for *a*-P and *a*-As. (C) Information (or Shannon) entropy of dihedral-angle distributions (in bits) for *a*-P and *a*-As. Here, the entropy quantifies the breadth and uniformity of the dihedral-angle distribution, reflecting the configurational diversity of local structures. (D,E) Counts of representative cluster fragments in *a*-P and *a*-As, respectively. Error bars denote uncertainties derived from ten independent simulations for each material, with full details provided in the Methods section. No error bars are shown for the information entropy, as the associated uncertainty would not be discernible within the scale of the plot.

Figure 5B shows that the population of large rings (*n* > 10) in *a*-P increases with increasing pressure. This is consistent with earlier studies, suggesting that cage-like motifs tend to open under compression, transforming into extended ring structures.^15^ In contrast, the number of large rings in *a*-As remains relatively stable with increasing pressure, exhibiting minimal change, even at elevated pressures (Figure 5B). To analyze this difference, we used the information entropy H^62^ to quantify the structural diversity encoded in the dihedral-angle distribution, which is given as

$$H=-\int \rho \left(\phi \right)\log_{2}\rho \left(\phi \right)d\phi$$ 1

where ρ­(*ϕ*) is the probability density of the dihedral angle, and the logarithm is taken with base 2, suggesting that H is measured in units of bits. A similar concept was proposed by Schwalbe-Koda et al., who used the information entropy to quantify the completeness of atomistic data sets.^63^ For *a*-P, the increase in the number of extended rings with pressure coincides with an increase in the dihedral-angle entropy (Figure 5C), indicating an increase in configurational diversity of the dumbbell shapes. This suggests that, at higher pressure, the structural flexibility of *a*-P increases, enabling the formation of extended rings. For *a*-As, the dihedral-angle entropy is already high at standard pressure, and both the number of extended rings and entropy remain practically unchanged under applied pressure (Figure 5C). Thus, changes in the ring statistics might be rationalized by the underlying dihedral-angle distributions, but it is clear that the underlying mechanism is different for *a*-As than for *a*-P.

Looking at the evolution of cluster fragments under pressure (Figure 5D,E), the contrasting behavior between *a*-P and *a*-As becomes even more apparent. In *a*-P, the counts of the representative fragment clusters remain relatively constant up to moderate pressures and then they reduce at higher pressures, whereas in *a*-As, the abundance of the main analogous cluster fragments, i.e., As2­[As3]­As2 and As3]­As2­[As3, increases under compression. This opposite behavior aligns closely with the changes in 5-membered ring counts in both systems: fewer 5-membered rings with high pressure in *a*-P restricts the formation of these compact clusters, while their increase in *a*-As with increasing pressure facilitates cluster assembly (Figure 5A). Moreover, the enhanced diversity of the dihedral-angle distribution in *a*-P results in a decreased probability of small dihedral angles, which are key to cluster formation, as discussed before (Figure 4D). As a result, cluster formation is suppressed in *a*-P at high pressures. The observed structural evolution under pressure emphasizes unexpected contrasting changes of MRO between these two chemically similar, yet structurally distinct, amorphous materials.

### Origin of the FSDP

As for *a*-P, the structure factor, *S*(*Q*), of *a*-As exhibits a pronounced FSDP (Figure 1C), a well-established signature of MRO in amorphous materials that has previously been linked to the spatial distribution of voids.^9,64^ To further investigate its microscopic origin, we examined the pressure dependence of the FSDP, alongside structural features associated with voids (Figure 6). Figure 6A shows representative atomic structures of *a*-As and *a*-P, with voids visualized as semitransparent purple and blue regions, respectively. These voids reveal the heterogeneous spatial arrangement of free volume and the distinct structural differences between *a*-As and *a*-P. Specifically, *a*-As shows a local layered arrangement in its void structure, reminiscent of the characteristic layering in crystalline black As, which consists of puckered layers held together by van der Waals interactions.^57^ This structural similarity between amorphous and crystalline As has also been highlighted in previous studies through comparable optical-reflection spectra, which indicate similar coordination environments and bonding arrangements.^65^

**Figure 6:**
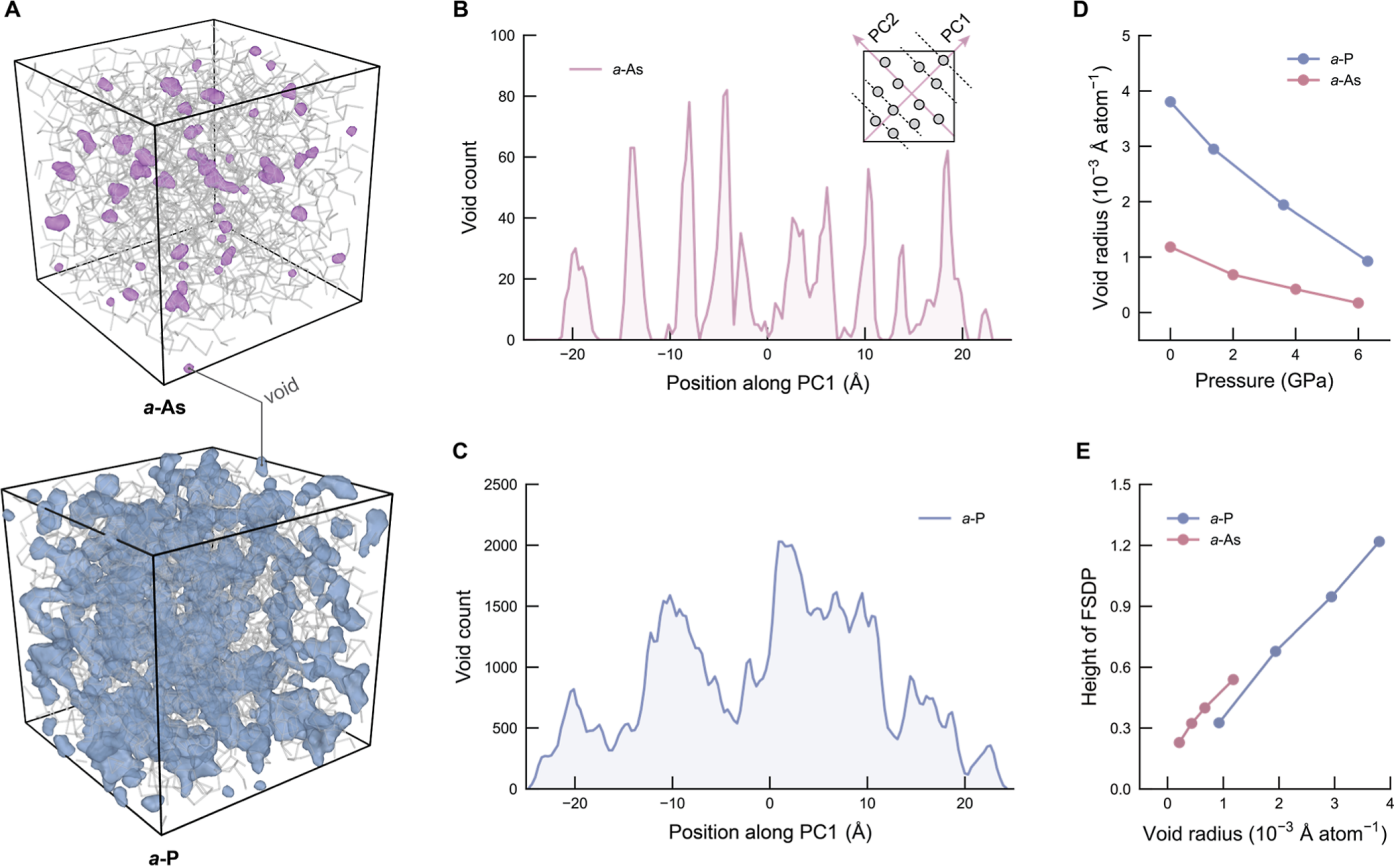
Correlation between voids and FSDP intensity. (A) Atomic structures of *a*-As (top) and *a*-P (bottom), with voids visualized as semitransparent purple and blue regions, respectively. The atomic coordinates of *a*-P were obtained from the literature.^15^ It shows that the void region in *a*-P is much bigger than that in *a*-As. (B) Spatial distribution of voids in *a*-As projected along the first principal-component (PC1) axis obtained from principal-component analysis (PCA) on the void coordinates. The inset provides a schematic of PCA. (C) Same but for *a*-P. (D) Comparison of the average equivalent void-radius change under pressure between *a*-As and *a*-P. The equivalent void radius is a normalized metric calculated by determining the radius of a hypothetical sphere whose volume equals the sum of all void clusters, then averaging this value over the total number of atoms in the system (eq 2). (E) Variation of FSDP height with average equivalent void radius, as an implicit function of pressure, showing a strong linear correlation.

To better understand this behavior, we applied principal-component analysis (PCA) to project the spatial distribution of voids onto a new coordinate system, where each axis is called a principal component, as illustrated in the inset of Figure 6B. Specifically, the first principal component (PC1) captures the primary spatial direction along which the void density varies most significantly. The distribution of void volumes along PC1 suggests that *a*-As exhibits an anisotropic, layered arrangement of voids, characterized by distinct, directional peaks in the void count (Figure 6B). We note that the importance of layer-like structural features has been pointed out in an earlier modeling study.^66^ In contrast, the void distribution in *a*-P appears more isotropic, with voids more evenly and uniformly dispersed throughout the structure, resulting in a relatively flat profile along PC1 (Figure 6C). The distributions of voids along the second and third principal components (PC2 and PC3) are shown in Figure S10.

Moreover, we quantified the pressure dependence of the average equivalent void radius, which is defined by

2 $$r={\left(3V/4\pi \right)}^{1/3}/N$$

where *V* is the sum of all void cluster volumes and *N* denotes the number of atoms in a system. In both materials, the equivalent void radius decreases with increasing pressure (Figure 6D). The (implicit) pressure dependence of the FSDP height exhibits a strong linear correlation with the equivalent void radius, with similar slopes observed in the two systems (Figure 6E). Together, these results support an interpretation that the FSDP arises from voids in *a*-As (and *a*-P), which become increasingly disrupted under compression.

## Discussion

The present study has provided new fundamental insight into the nature of medium-range structural order in amorphous materials. Specifically, we have shown that, although both P and As adopt similar, 3-fold-bonded local atomic environments, their amorphous modifications exhibit markedly different medium-range structural order. At ambient pressure, *a*-As contains many large rings (*n* > 6), which are practically absent in *a*-P. We have shown that this difference in MRO can be directly correlated with a difference in the respective dihedral-angle distributions, which, in turn, reflect the particular chemical-bonding nature: the greater torsional flexibility of bonds in *a*-As allows more extended ring structures to form. Under compression, *a*-P forms increasingly large ring structures with fewer compact clusters,^15^ whereas *a*-As maintains the diversity of large-ring motifs and fragments. Collectively, these results highlight how flexibility in dihedral angles can influence medium-range structural diversity in the amorphous state.

We emphasize the striking contrast in medium-range order between the chemically homologous systems *a*-P and *a*-As. Both elements have s^2^p^3^ valence configurations and might therefore be expected intuitively to form 3-fold coordinated continuous-random-network (CRN) structures, akin to the 4-fold coordinated CRN that approximates amorphous silicon.^67^ However, our results for the dihedral-angle distributions (Figure 4B) emphasize a qualitative difference in the type of amorphous structures for As and P. The more uniform dihedral-angle distribution for *a*-As is indeed consistent with its structure being well approximated as a 3-fold coordinated CRN model, as discussed in the 1970s in ref 68 (and refined in ref 69). In contrast, the very nonuniform dihedral-angle distribution for *a*-P is not consistent with the structure being CRN-like; rather it is more of a molecular-cluster glass.^15,70^ The structure of *a*-As is also a bridge to the very different atomic structure of *a*-Sb: ab initio MD simulations revealed that the nearest-neighbor coordination in this material is not mainly 3-fold, as in As and P—instead, the local environments in *a*-Sb are primarily defective-octahedral, with 4-, 5-, and 6-fold coordination.^71^

In terms of modeling methodology, our study has demonstrated how automated MLIP fitting workflows enable the efficient generation of interatomic potentials for bulk amorphous systems: in the present case of *a*-As, we have been able to generate a bespoke MLIP model that reproduces the experimental structure factor at a total computational cost of less than 50,000 CPU core hours. The combination of RSS workflows with iterative MD refinement proved crucial for achieving improved predictions of structural factors of *a*-As. Beyond elemental systems, based on pilot studies for ternary chalcogenide memory materials^32^ and a recent investigation of amorphous Na–P structures involving autoplex as one component of the training data generation,^72^ we anticipate that MLIP-driven studies of multicomponent amorphous systems will benefit from increasingly automated workflows and general protocols.

We note that the current RSS workflow does not yet extend to surfaces, heterogeneous, or reactive systems. For such future work, the RSS-generated potential may serve as a reasonable starting point, from which one could iteratively refine the model through targeted MD sampling of relevant environments—such as defects, interfaces, or reaction pathways—combined with active-learning-based selection of informative configurations, as also discussed in ref 32. Specific examples in the context of the present work could be an extension to few- and monolayer arsenic (arsenene),^73,74^ including possible defects and disordered regions within it, or the synthesis of amorphous and crystalline boron arsenide (BAs) nanosheets.^75^ All in all, applying automated MLIP generation broadly across different chemical families promises new insights into structure–property correlations in amorphous solids, thereby offering a generalizable strategy to uncover how atomic-level interactions define medium-range order and macroscopic properties.

## Methods

### MLIP Generation with Automated Workflows

The autoplex framework (version 0.0.7, available at https://github.com/autoatml/autoplex) was used to generate the RSS data set in a largely automated manner.^32^ In each iteration, 10,000 random structures containing 6 to 24 atoms (even numbers) per cell were generated. Among them, 20% were constrained to possess between 2 and 8 symmetry operations, while the remaining 80% were generated without any symmetry constraints, to enhance the diversity of the initial structural pool. To ensure that the RSS procedure adequately samples configurations relevant to high-pressure polymorphs, external pressures were applied during the generation stage. These pressures were assigned according to an exponential distribution, following the approach introduced by ref 37, with a scale factor of 10 GPa and a width of 0.2, thereby providing coverage of both ambient- and high-pressure regions of configuration space (Figure S11).

Energies, forces, and stresses of the RSS-generated structures were obtained from DFT single-point computations with VASP,^76,77^ managed by atomate2 ^78^ through interfaces in autoplex.^32^ The DFT computations employed the projector augmented-wave (PAW) method^77,79^ and the r^2^SCAN functional,^44^ a regularized form of SCAN^80^ with improved numerical stability. The plane-wave energy cutoff was 600 eV. A *k*-point spacing of 0.2 Å^–1^ was used to sample reciprocal space. Electronic self-consistency was converged to a tolerance of 1 × 10^–7^ eV, and a Gaussian smearing width of 0.01 eV was used for partial occupancies. After each iteration, 100 data points were generated, with 90% used as the training set and the remaining 10% as the test set. The training and test sets for each iteration are cumulative, incorporating data from all previous iterations.

In addition to RSS, autoplex was also used to manage high-throughput DFT calculations for evaluating the energy of structures sampled from MD trajectories to refine the potentials. In this case, a workflow composed of the DFTStaticLabelling and collect_dft_data functions within autoplex was employed to generate a formatted data set ready for MLIP training. For the larger cells of 216 atoms, we used Γ-point calculations, with all other parameters kept the same as those used for the RSS data set. Although GAP and MACE are different MLIP fitting frameworks, we note that all GAP-sampled configurations were fully re-evaluated at the DFT level before MACE training.

### Training MLIPs

In this work, iterative RSS was driven by GAP models.^25,35,36^ Except where noted, our GAP setup and hyperparameter choices match that of the earlier P-GAP-20 model.^48^ We disabled the “R6” baseline pair potential that had been used in ref 48, while retaining both the two-body term and the many-body SOAP term. The number of sparse points for the two-body term was 15 in both models, while that for SOAP was reduced from 8000 (ref 48) to 3000 here, enabling faster evaluations at a speed of approximately 2 min per structure on a single CPU core. The weights for energies, forces, and stresses were assigned automatically within autoplex, based on the structures’ distances from the energy convex hull (see also ref 33).

We used the MACE framework^38^ to retrain a model on the pure RSS data set and fit refined data sets that were iteratively augmented with MD structures (Figure 1A). For training MACE models at various DFT levels of theory, we used the same hyperparameters. Each model employed two message-passing layers with 128 channels. A correlation order of 3 was chosen, and spherical harmonics were set up to degree 3. The cutoff radius was 6 Å, resulting in a total receptive field of 12 Å per atom. We also tested a range of cutoff radii from 4 to 8 Å and found that 6 Å yielded the lowest energy errors when predicting the energies and structure of *a*-As (Figure S12). The models were trained using the Huber loss function^81^ for energies, forces, and stresses. Double precision was used throughout, and the maximum number of training epochs was set to 2000. We trained all MACE models with the mace-torch package on an NVIDIA A100 80GB PCIe GPU, with the final complete training taking two and a half hours, resulting in an estimated GPU-only energy consumption of ∼0.75 kWh.

It should be noted that we did not apply any explicit term to MACE to capture the long-range dispersion interactions present at the r^2^SCAN + rVV10 level; instead, the model learns dispersion implicitly from the DFT reference data, with only the contributions falling within the receptive field being represented. We further verified the transferability of this approach by evaluating model performance across increasingly large amorphous supercells (Figure S13).

### MD Simulations

ML-driven MD simulations were performed with LAMMPS.^82^ The structure of *a*-As at ambient conditions was obtained via a melt-quench simulation protocol. An initial cubic cell containing 2000 atoms (comparable to the 1984 atoms used previously in models of *a*-P^15^), at an experimental density of 4.74 g cm^–3^,^40^ was created using buildcell^34,83^ with hard-sphere potentials to ensure reasonable interatomic distances. The structures were first equilibrated at 3000 K for 30 ps in the *NVT* ensemble to randomize atomic positions and remove structural ordering. This was followed by a melting simulation at 1500 K for another 30 ps. The system was then cooled from 1500 to 300 K at a rate of 10^12^ K s^–1^ (corresponding to 1.2 million MD steps). A comparison of different quench rates, and their effects on the resulting structures, can be found in the Supporting Information. After reaching 300 K, the system was annealed for 50 ps in the *NVT* ensemble, followed by an additional 50 ps of annealing in the *NPT* ensemble using a Nosé–Hoover thermostat and barostat.^84,85^ The structure factor was calculated from the final 10 ps of the trajectory (200 snapshots) and compared with experimental data. Subsequently, compression simulations were performed at 300 K by gradually increasing the pressure from ambient conditions up to 6 GPa at a compression rate of 0.02 GPa ps^–1^, consistent with that used in the previous study on *a*-P^15^ for a fair comparison. The time step in all MD simulations was 1 fs.

### Sensitivity and Uncertainty Evaluation

Additional tests evaluating the sensitivity of the simulated structures to the quench rate (including a lower quench rate of 10^11^ K s^–1^), the *NPT* annealing time at 300 K (extended from 50 to 500 ps), and the system size (2000 versus 4000 atoms) are provided in the Supporting Information (Figure S14). These analyses confirm that the structural features are robust with respect to the chosen simulation parameters.

To quantify the uncertainty of the MLIP predictions, we performed ten independent melt-quench-compression simulations for 2000-atom cells of As, each initiated from different random configurations and velocity seeds. The resulting structure factors give an FSDP height of 0.56 ± 0.01. The quantitative characterization of MRO (Figure 5) under compression was obtained by averaging the results over ten independent simulations, with the associated uncertainty estimated from the variance across these samples. For the uncertainty of energy predictions, we additionally carried out melt-quench simulations for 512-atom and 1024-atom cells, extracted representative structures from the final 10 ps at 300 K, and re-evaluated their energies with DFT at the r^2^SCAN level. Across 20 samples, the mean energy RMSE is 7.9 ± 1.3 meV atom^–1^.

To estimate the uncertainty in the MRO of *a*-P (Figure 5), we did not regenerate the new melt-quench trajectories from scratch, as previous studies required simulation times on the order of 10 ns, which would be prohibitively expensive with GAP. Instead, we performed ten independent compression simulations, each starting from a different velocity distribution and preceded by a 50 ps equilibration at 300 K and used these runs to determine the corresponding uncertainties.

### Dihedral Angles

In this work, we analyze dihedral angles for pairs of 3-fold-bonded atoms, taking into account those atoms’ neighbor environments rather than calculating separate dihedral angles for all individual bonds. To calculate the dihedral angles in the dumbbell structural motifs (Figure 4A), we first computed the sum of bond vectors from each central atom of connected trigonal units (*c* and *c*′) to its two neighboring atoms, yielding two directional vectors, **s** and **s′**. The dihedral angle, ϕ, was then calculated as the angle between the planes spanned by {**s**, **r**_*cc*′_} and {**s′**, **r**_*cc*′_}, where **r**_*cc*′_ is the vector from atom *c* to atom *c*′. Note that central atoms which do not have three neighbors (considered to be coordination defects in *a*-As) are discarded in this analysis. To quantify the structural diversity encoded in the dihedral-angle distribution, we evaluated the information entropy H (also known as the Shannon entropy) using its standard definition from statistics and information theory^62^ (eq 1).

### Void Analysis

We used a grid-based spatial search method to identify voids. In this method, a “void” grid was defined as a region of space where no atomic center existed within a specified cutoff distance. For each material, the cutoff distance was chosen to correspond to the first minimum of its radial distribution function: viz. 2.9 Å for *a*-As and 2.4 Å for *a*-P. A uniform Cartesian grid was generated within the simulation cell, with the grid spacing set to one-10th of the chosen cutoff distance. Each grid point was then compared against the set of all atomic positions, including periodic images, with a cKDTree search;^86^ points with a nearest-atom distance greater than the cutoff were labeled as “void voxels”. These void voxels were subsequently clustered via the DBSCAN algorithm using a clustering radius of 1.2 × grid spacing and a minimum cluster size of five points (discarding clusters smaller than five points).^87^ Each cluster represented a contiguous free-volume region, with its volume equal to the number of voxels in the cluster multiplied by the voxel volume. The combined volume of all retained clusters was converted into an average equivalent spherical radius (eq 2). A sensitivity test of void-algorithm parameters, including grid spacing, cutoff distance, and minimum cluster size, is presented in the Supporting Information (Figure S15).

Based on the recorded coordinates of each void voxel, PCA was applied to determine the direction along which the void spatial arrangement exhibits the greatest variance, namely the first principal component (PC1) axis.^88^ More specifically, given a set of *N*_*v*_ voxel positions ${\left\{x_{i}\right\}}_{i=1}^{N_{v}}$ in 3D space, the data were first mean-centered

3 $$x_{i}^{c}=x_{i}-\mathrm{x̅}$$

where x̅ is the mean position of all void voxels. The covariance matrix was then computed as

4 $$C=\frac{1}{N_{v}-1}\sum_{i=1}^{N_{v}}x_{i}^{c}{\left(x_{i}^{c}\right)}^{T}.$$

The first principal component (PC1) was obtained as the eigenvector **v**_1_ corresponding to the largest eigenvalue of **C**. Each voxel position was then projected onto PC1 via

$$p_{i1}=\left(x_{i}-\mathrm{x̅}\right)\cdot v_{1}$$ 5

where *p*_*i*1_ denotes the coordinate of the *i*-th void along the PC1 axis. The coordinates along the second and third principal components (PC2 and PC3) can be similarly obtained by projecting **x**_*i*_ onto the corresponding eigenvectors.

### Assessment of Structural Diversity in the RSS Data Set

The structural diversity of the RSS data set was analyzed using SOAP descriptors, combined with PCA and a convergence metric based on the Jensen–Shannon (JS) divergence.^89^ Atom-wise SOAP descriptors were computed for every configuration using the DScribe implementation^90^ with settings of *n*_max_ = 8 and *l*_max_ = 8, a Gaussian smearing width of 0.5 Å, and a cutoff radius of 5 Å.

To visualize the diversity of local atomic environments, atom-wise SOAP vectors from the RSS data set and the amorphous compression trajectory were embedded in a shared two-dimensional PCA space (Figure S16A). It is seen that the local atomic environments in the RSS configurations span a considerably broader region of the SOAP space, whereas those in the amorphous structure occupy a much narrower manifold nested within the RSS distribution, suggesting that small-cell RSS sampling is sufficient to capture the relevant diversity of local environments in *a*-As.

To assess how the structural diversity of the RSS data set saturates with increasing the training data, we incrementally accumulated structure-level SOAP descriptors, constructed via the “inner” averaging scheme to yield one SOAP vector per structure, such that the partial data set after iteration *i* contained all configurations generated up to that iteration. For each such iteration-accumulated data set, the corresponding SOAP vectors were projected onto the fixed PCA basis obtained from the complete RSS data set. The distribution of the first principal component was estimated using one-dimensional kernel density estimation (bandwidth = 0.1), and the JS divergence was computed between the data from iteration *i* and that from iteration *i* – 1 (Figure S16B). It is seen that the JS divergence exhibits an overall decreasing trend with the number of iterations and reaches a plateau after 13 iterations, indicating that the SOAP descriptor distribution becomes saturated and that subsequent RSS iterations contribute only marginal additional structural diversity.

## Supplementary Material



## Data Availability

Data supporting this work, including raw data and Python notebooks to reproduce the plots, are available via GitHub at https://github.com/autoatml/papers-liu-as. This work used the autoplex software (v0.0.7), which is openly available at https://github.com/autoatml/autoplex. The code for the void analysis is openly available at https://github.com/vldgroup/voidkit.

## Notes

Supporting Information with computational and bonding analyses, Figures S1–S16, and Table S1 (PDF)
