# Supplementary material for: Medium-Range Structural Order in Amorphous Arsenic
Source: J Am Chem Soc. 2026 Feb 26;148(9):9400–12. doi: 10.1021/jacs.5c18688 (PMC12983317; doi:10.1021/jacs.5c18688)
Supplement: Supplementary file 1 [file ja5c18688_si_001.pdf]

Supporting Information

Medium-Range Structural Order in Amorphous Arsenic

Yuanbin Liu[1], Yuxing Zhou[1], Richard Ademuwagun[1], Luc Walterbos[2,3], Janine George[2,3], Stephen R. Elliott[4], Volker L. Deringer[1]*

[1]*Inorganic Chemistry Laboratory, Department of Chemistry, University of Oxford, Oxford OX1 3QR, UK*

[2]*Materials Chemistry Department, Federal Institute for Materials Research and Testing (BAM), Unter den Eichen 87, 12205 Berlin, Germany*

[3]*Institute of Condensed Matter Theory and Solid-State Optics, Friedrich Schiller University Jena, Max-Wien-Platz 1, 07743 Jena, Germany*

[4]*Physical and Theoretical Chemistry Laboratory, Department of Chemistry, University of Oxford, Oxford OX1 3QZ, UK*

* volker.deringer@chem.ox.ac.uk

This PDF file includes supplementary text, Figures S1–S16, and Table S1.

Supplementary Text

For chemical-bonding analysis, we computed crystal orbital Hamilton population (COHP)[S1] and crystal orbital bond index (COBI)[S2] data with the program LOBSTER[S3,S4] and analyzed the output using LobsterPy.[S5,S6]

LOBSTER analysis is based on a projection of the DFT wavefunctions onto an auxiliary basis of localized, atom-centered orbitals (4s and 4p on each As atom, and 3s and 3p on each P atom). For arsenic, plotted alongside the electronic densities of states (DOS) in Figure S5, the –COHP curves quantify bonding and antibonding interactions, while COBI provides information on the bond order across different energy levels. Gray As shows more metallic properties than black As and *a*-As, which has been attributed to its structural relationship with the simple cubic type.[S7] Comparing *a*-As with the crystalline phases, the electronic structure of *a*-As appears to share the most similarities with that of the black-As phase. This is again in line with previous observations.[S8] However, whereas black As has a small bandgap (0.3 eV), our *a*-As model appears to feature states at the Fermi level. We think that this is likely caused by defect states due to under-coordinated As atoms, consistent with an earlier study of the electronic properties of *a*-P.[S9] Bond strength dependence on the dihedral angle in *a*-As (Figure S6) and *a*-P (Figure S8) was quantified by –ICOHP. In *a*-As, the bond strength exhibits a weak dependence on the dihedral angle, whereas in *a*-P, the dihedral angles show a clear preference near ±55°.

The electronic-structure analyses were performed for both As and P at the r^2SCAN+rVV10 level of theory,[S10] with a plane-wave energy cutoff of 600 eV. For crystalline structures of As, a *k*-point spacing of 0.2 $Å^{-1}$ was employed, while for *a*-As and *a*-P, we used Γ-point calculations. For the experimentally known crystalline As phases (gray and black), the initial structures were taken from the Materials Project.[S11] For the hypothetical modifications (yellow, violet, and fibrous) of As, the initial structures were generated by substituting As into the corresponding P structures.[S9] (We note that other crystalline structures for As have recently been discussed based on experiments[S12] and computation,[S8,S13] but we do not include those in Figure S5.) All relevant, initial crystalline structures of As, including hypothetical ones, were subsequently fully relaxed using the r^2SCAN+rVV10 functional. The amorphous structure of As contained 500 atoms and was generated by relaxing the melt–quench configuration with our MLIP. The structure of *a*-P with 248 atoms was taken from Ref. S9, in which it was labeled as **2**. The nearest-neighbor bonds were identified using the `COHPgenerator` keyword in LOBSTER, with the cut-off for bond detection set to 2.4 Å for P and 2.9 Å for As.

Supplementary Figures

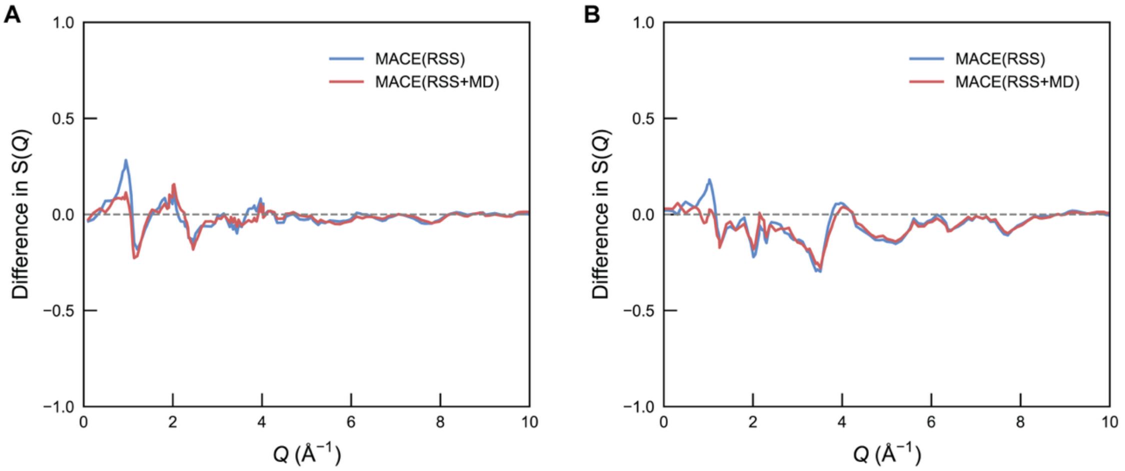

Figure S1: Structure-factor differences between experiments and two MACE models. (**A**) Experimental data from Smith et al.[S14] (**B**) Experimental data from Bellissent and Tourand.[S15] The curves show the difference obtained by subtracting the simulated values from the experimental data for each model. It shows that the additional MD-based refinement primarily increases the intensity of the predicted FSDP, whereas the remainder of the curve is essentially unchanged compared to that predicted by the purely RSS-trained model.

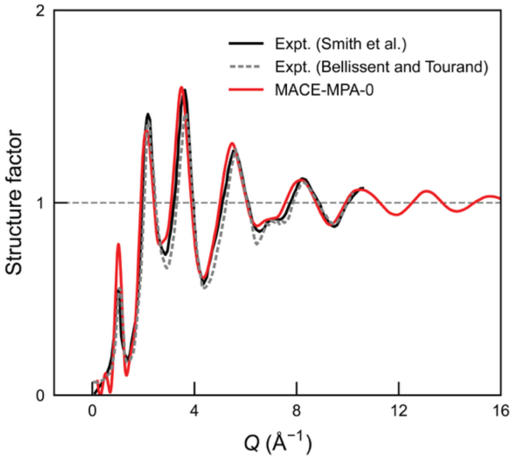

Figure S2: Benchmarking the MACE-MPA-0 foundation model against experimental data. The structure factor of *a*-As calculated using the MACE-MPA-0 model[S16] shows qualitative agreement with experimental results from Smith et al.[S14] as well as Bellissent and Tourand.[S15] However, the model exhibits quantitative discrepancies, notably overestimating the intensity of the first sharp diffraction peak (FSDP).

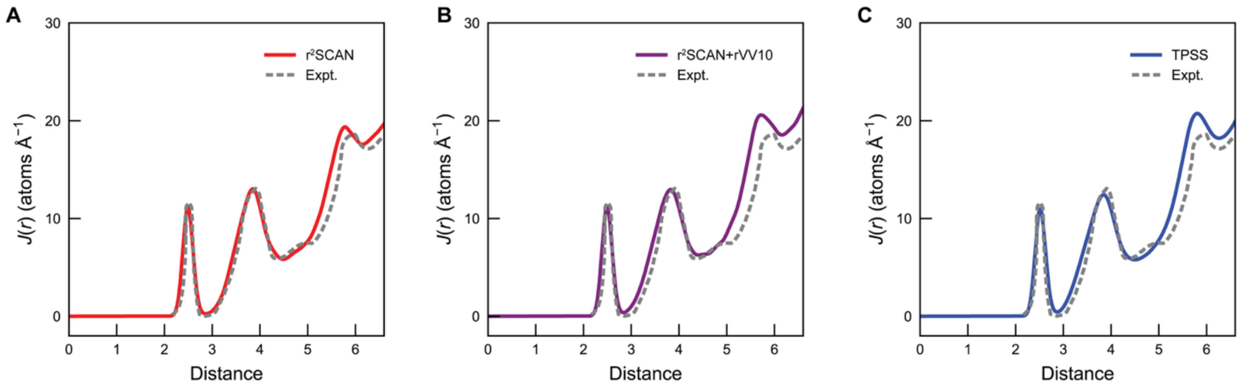

Figure S3: Benchmarking meta-GGA functionals via radial distribution functions of amorphous arsenic. Radial distribution functions, $J(r)$, calculated with different meta-GGA functionals are compared with experimental data.[S17] We show results based on: (**A**) r^2SCAN; (**B**) r^2SCAN+rVV10; and (**C**) TPSS. All functionals reproduce the experimental trends, with r^2SCAN showing the closest agreement and further validating the accuracy of our choice of functional.

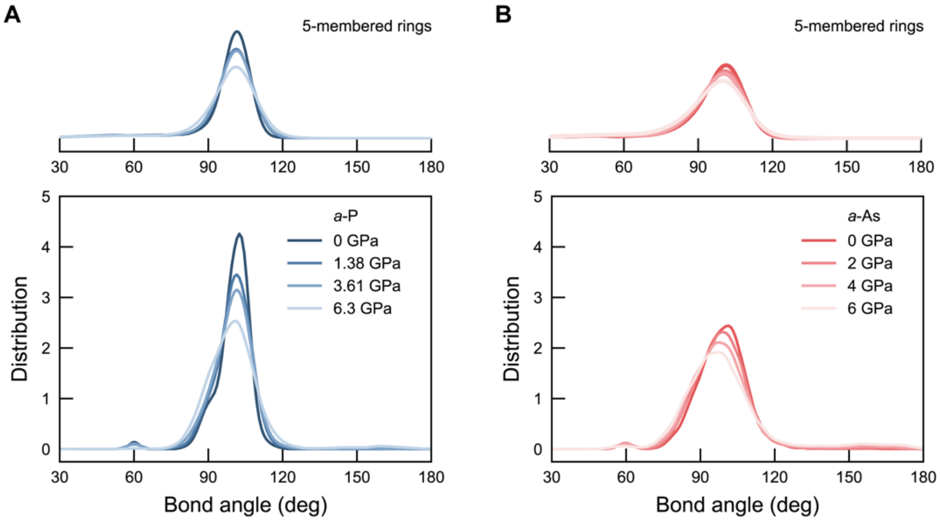

Figure S4: Bond-angle distribution as a function of pressure. (**A**) *a*-P and (**B**) *a*-As. Kernel density estimates at the top of each panel show the bond-angle distributions within 5-membered rings, while the lower panels display the probability-density functions of the total bond-angle distributions calculated for all atoms in the systems. Notably, the dominant peak in the total bond-angle distribution coincides with that of the 5-membered rings, suggesting that their similar angular preferences facilitate the formation of 5-membered rings in both *a*-P and *a*-As.

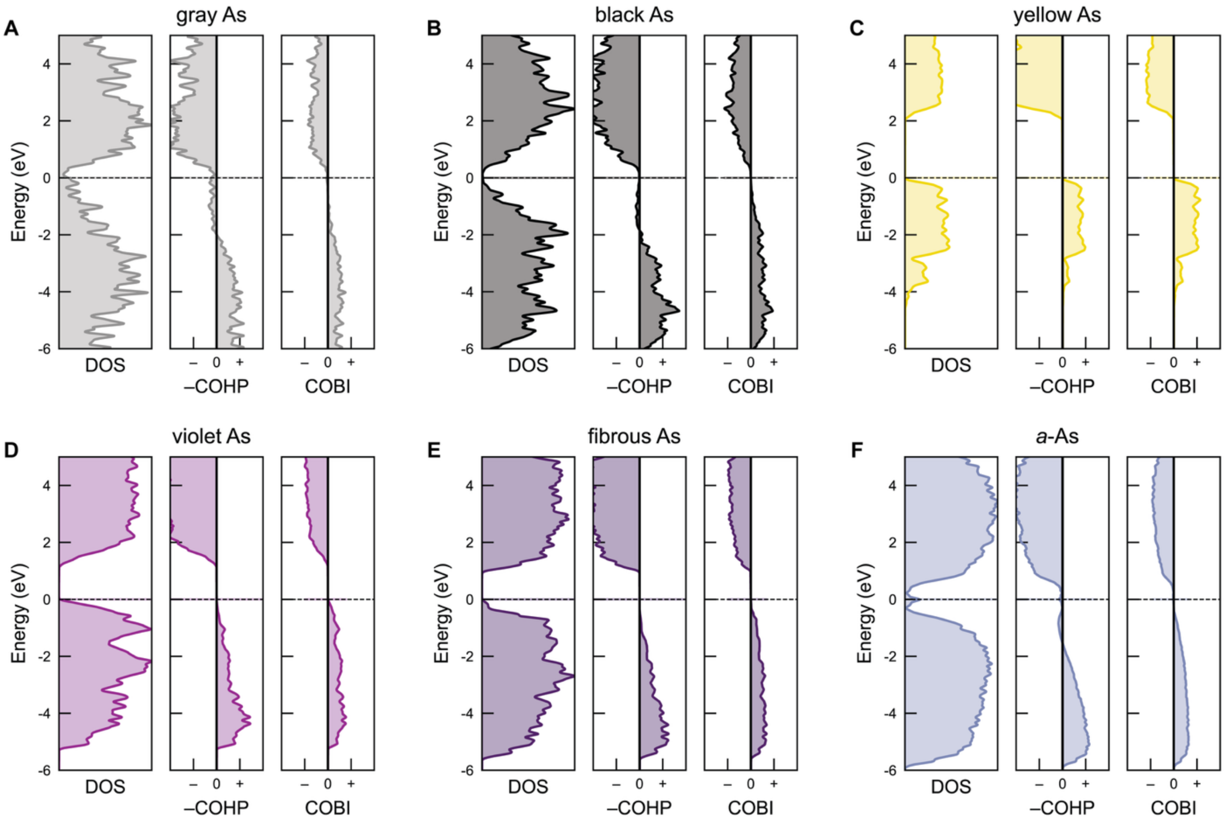

Figure S5: Electronic structure and chemical-bonding analysis of crystalline and amorphous As phases. Electronic density of states (DOS), projected crystal orbital Hamilton population (COHP), and crystal orbital bond index (COBI) plots of six relevant As phases: (**A**) gray As; (**B**) black As; (**C**) yellow As; (**D**) hypothetical "violet As" (derived from violet P by elemental substitution and subsequent relaxation); (**E**) as in (D) but for "fibrous As"; and (**F**) amorphous As (*a*-As). See the Supplementary Text on p. S2 for details.

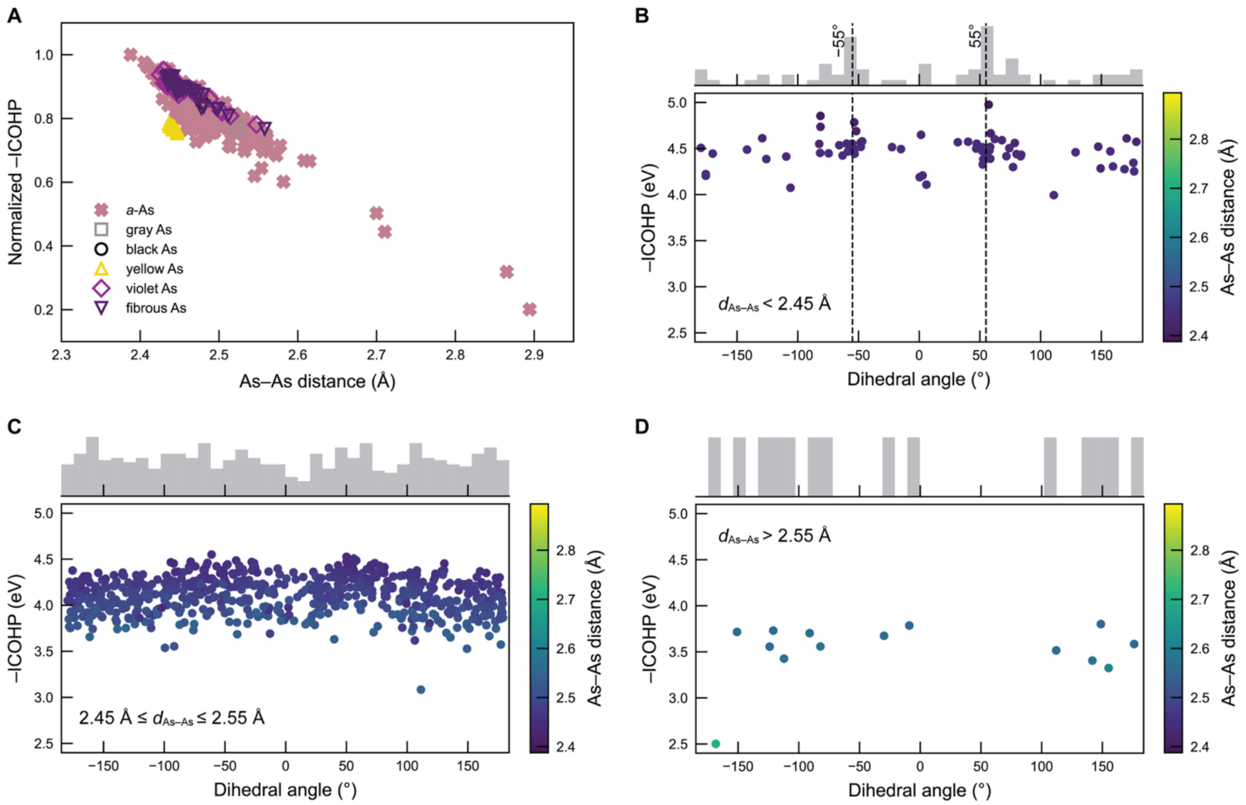

Figure S6: Structure and bonding in crystalline and amorphous As. (**A**) Bond length–bond strength relationship, where each symbol represents an As–As bond. The ICOHP values, taken to measure the bond strength, are normalized to the value for the strongest bond in the *a*-As network (–ICOHP = 4.95987 eV). Our analysis shows that the bond strengths of the nearest-neighbour interactions in *a*-As span a comparatively large range. The average bond energy (–ICOHP = 4.10231 eV) and bond length in *a*-As are roughly equal to the average of the bond energy and bond length in black As (–ICOHP = 4.20101 eV). (**B**–**D**) Dependence of bond strength on dihedral angle in *a*-As, distinguished by bond-length regime: short (B, $d_{\mathrm{As–As}} < 2.45$ Å), intermediate (C, $2.45 \leq d_{\mathrm{As–As}} \leq 2.55$ Å), and long (D, $d_{\mathrm{As–As}} > 2.55$ Å) As–As bonds. The color bar denotes the As–As bond distance, while the histograms show the statistical distribution of bond numbers. Short As–As bonds (B) show a preference for dihedral angles near ± 55°. By contrast, intermediate and long bonds (panels C, D) show no strong dependence of bond strength on dihedral angle. Generally, stronger bonds (more negative ICOHP values) are associated with shorter bond lengths, whereas the dihedral angle shows no strong correlation with the bond strength. The electronic-structure computations were performed using settings as described in the Supplementary Text (p. S2).

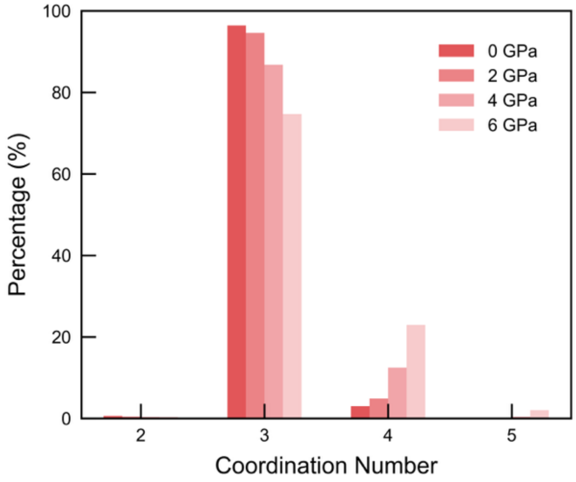

Figure S7: Coordination-number distribution of *a*-As under pressure. The proportion of 3-fold coordinated atoms decreases with increasing pressure, while the occurrence of over-coordinated environments (primarily 4-fold) becomes more prominent. The 2-fold coordination remains negligible for all pressures. A cutoff distance of 2.9 Å was employed to define coordination numbers.

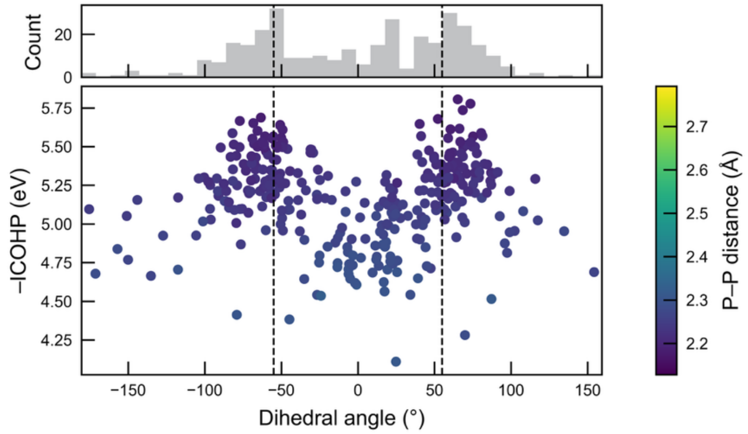

Figure S8: Chemical bonding in *a*-P. It shows the strong dependence of bond strength on dihedral angle in *a*-P. This is reflected in a preference for dihedral angles near ± 55°. In contrast, *a*-As exhibits little angular dependence (Figure S6C), consistent with softer bonding and larger angular flexibility.

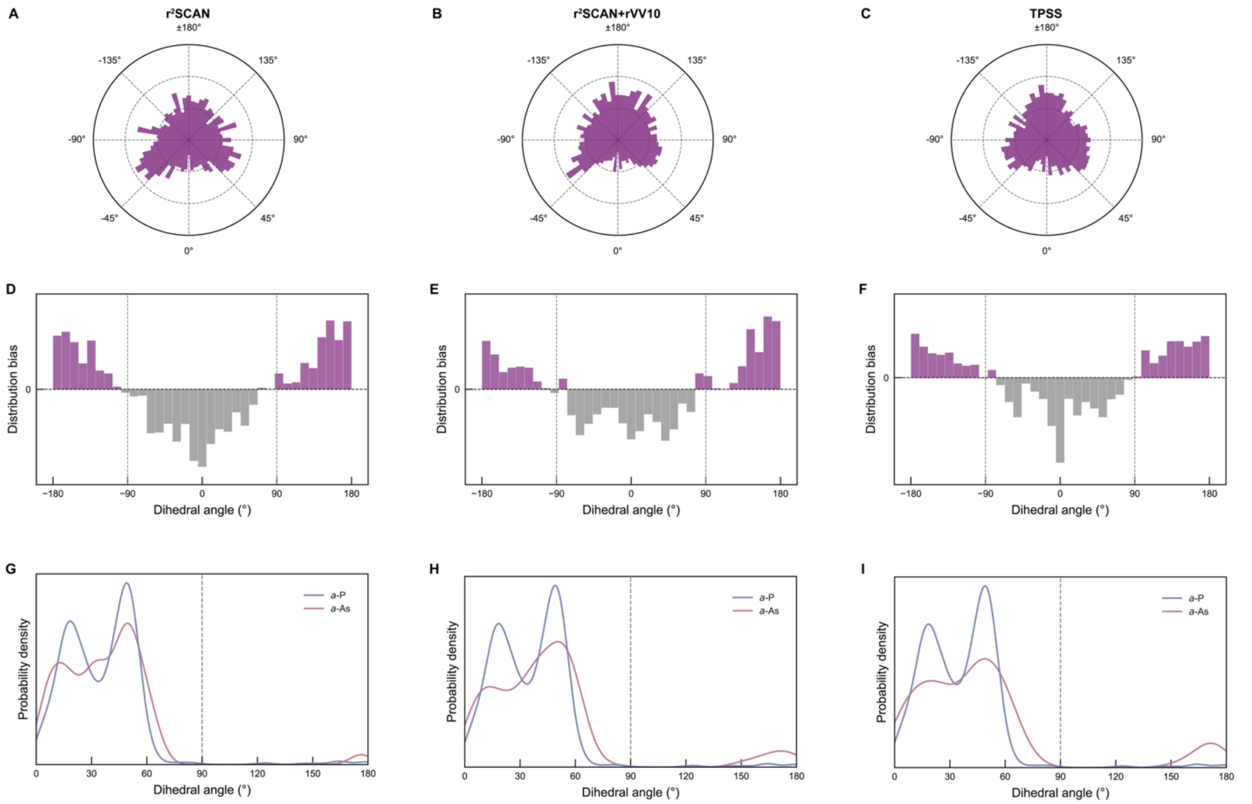

Figure S9: Dihedral-angle characteristics of models of *a*-As simulated using MLIPs trained with different functionals. (**A–C**) Polar histograms of dihedral-angle distributions for models of *a*-As obtained from MLIPs trained with r^2SCAN, r^2SCAN+rVV10, and TPSS, respectively. (**D–F**) Distribution bias in dihedral angles between large ($n > 6$) and small ($n \leq 6$) membered rings for the corresponding functionals. (**G–I**) Probability-density distributions of dihedral angles in $X_3]X_2[X_3$ fragment clusters, the most prevalent cluster type in *a*-As, for the corresponding functionals. It indicates that changing the functional does not affect the main conclusions regarding dihedral angles: the dihedral-angle distribution in *a*-As is broad; larger rings in *a*-As tend to comprise large dihedral angles (>90°); and dihedral angles in $X_3]X_2[X_3$ clusters are much more likely to be smaller than 90°.

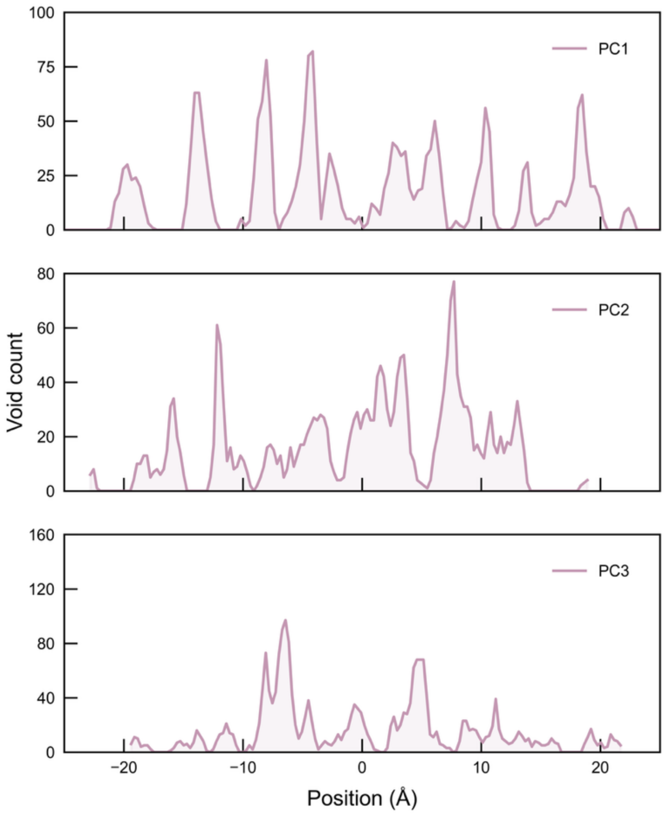

Figure S10: Spatial distributions of voids in *a*-As projected along principal-component axes. The three panels correspond to the distributions along the first (PC1), second (PC2), and third (PC3) principal components. This plot illustrates that peak patterns are more distinct along PC1.

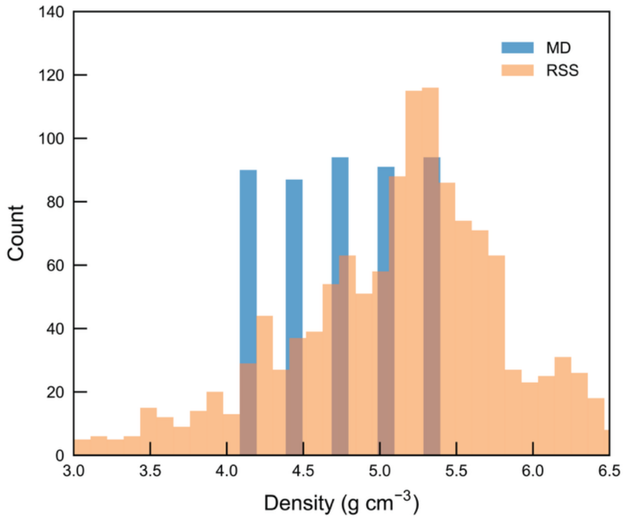

Figure S11: Density distributions of configurations sampled during the RSS stage (orange) and the MD stage (blue). During the RSS stage, external pressures were sampled from an exponential distribution with a scale factor of 10 GPa and an exponential width of 0.2, which produces configurations spanning a wide pressure range, including those above 6 GPa.

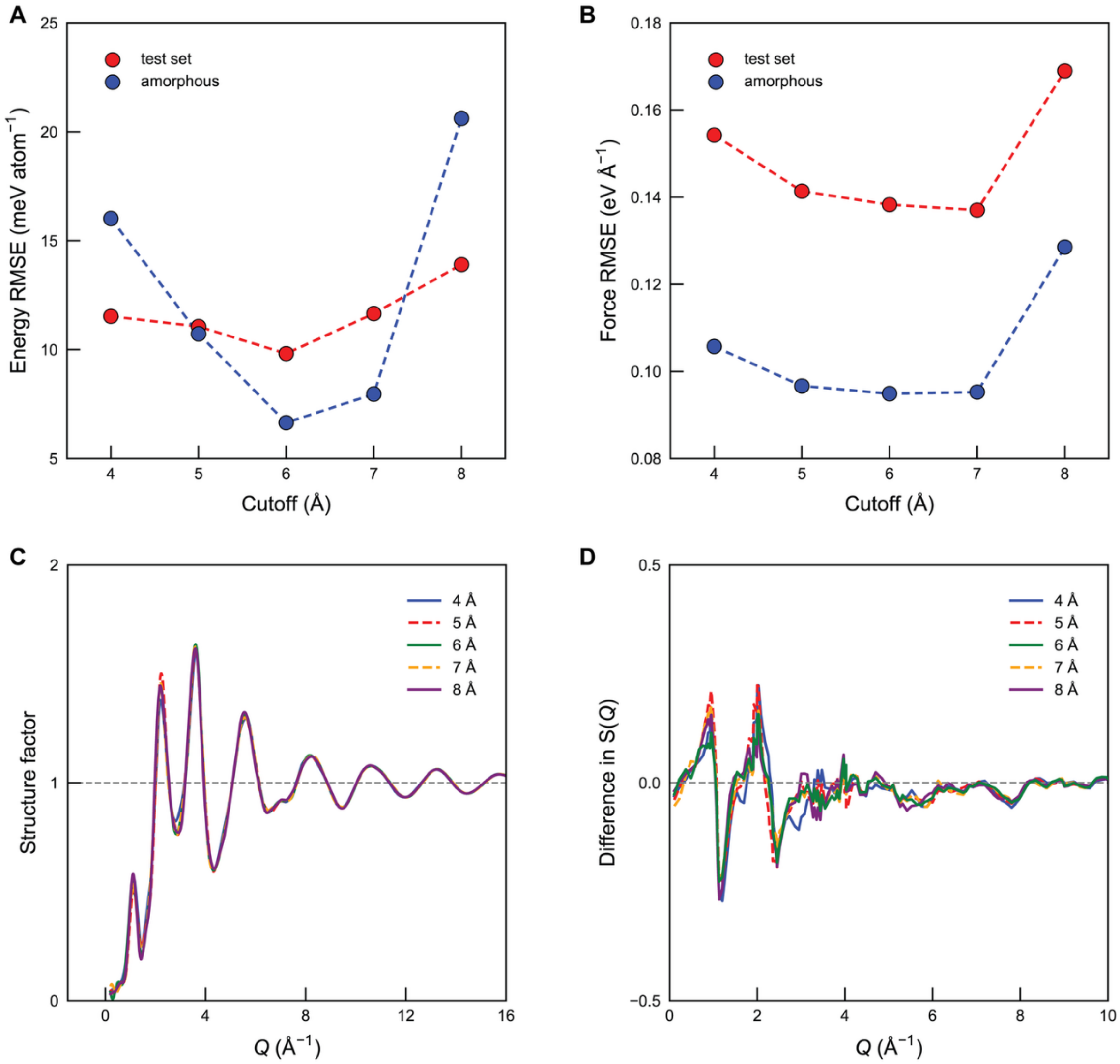

Figure S12: Effect of the cutoff radius on the accuracy of the MACE model. (**A**) Energy and (**B**) force RMSEs evaluated for two datasets: the test set, drawn from the same dataset used to train and validate the potentials, and the amorphous dataset, consisting of ten 1,000-atom amorphous configurations generated via a fast MACE-driven melt–quench simulation at 10^{13} K s^{-1} and labelled at the r^2SCAN level. (**C**) Structure factors obtained from different MACE models. (**D**) Differences between the experimental structure factor and those predicted by MACE models. All models were trained using identical training and validation datasets, with the cutoff radius being the only hyperparameter varied.

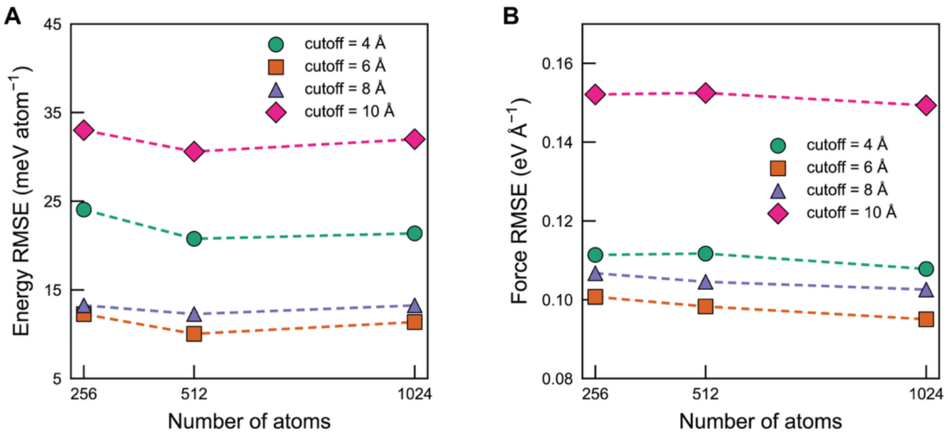

Figure S13: Transferability of MACE models of *a*-As across system sizes when trained on r^2SCAN+rVV10 reference data. For each system size, we performed a fast ML-driven melt–quench simulation at a cooling rate of 10^{13} K s^{-1}. From the final 10 ps of the 300 K annealing trajectory, ten configurations were extracted and recomputed at the r^2SCAN+rVV10 level for labelling energies, and the resulting MACE energy and force RMSEs were then evaluated against these reference data. (**A**) Energy RMSE and (**B**) force RMSE for amorphous systems containing 256 to 1,024 atoms, evaluated for models trained with different cutoff radii. Both quantities show only modest variations with system size, demonstrating good transferability to larger amorphous cells. Among the tested values, the 6 Å cutoff yields the best overall accuracy, suggesting that increasing the cutoff radius does not necessarily improve predictive accuracy of MACE models. All models were trained using identical datasets, with the cutoff radius being the only hyperparameter varied.

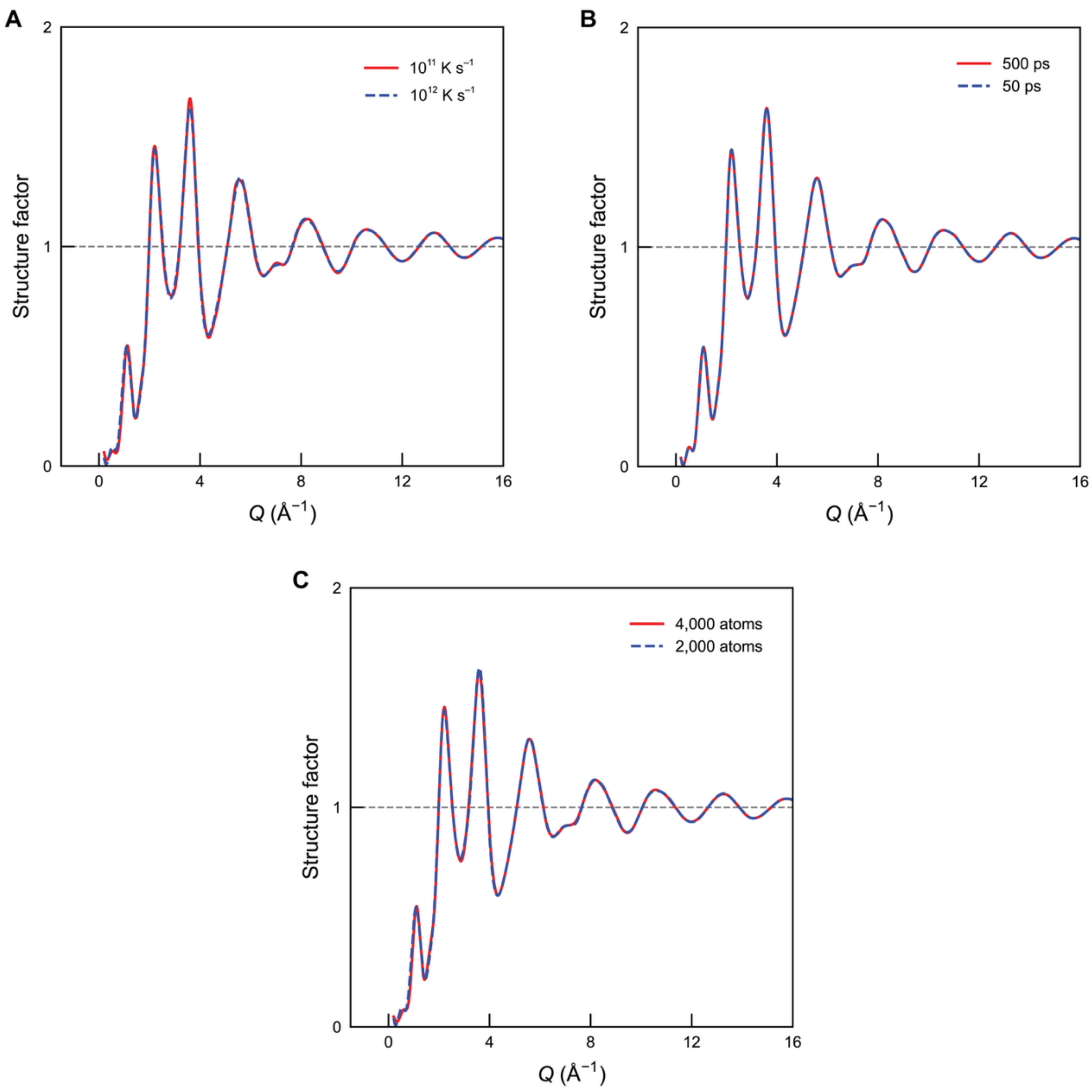

Figure S14: Sensitivity of the simulated structure factor to quench rate, annealing time, and system size. (**A**) Quench rates of 10^{11} K s^{-1} and 10^{12} K s^{-1}. (**B**) Annealing times at 300 K of 50 ps and 500 ps. (**C**) System sizes of 2,000 atoms and 4,000 atoms.

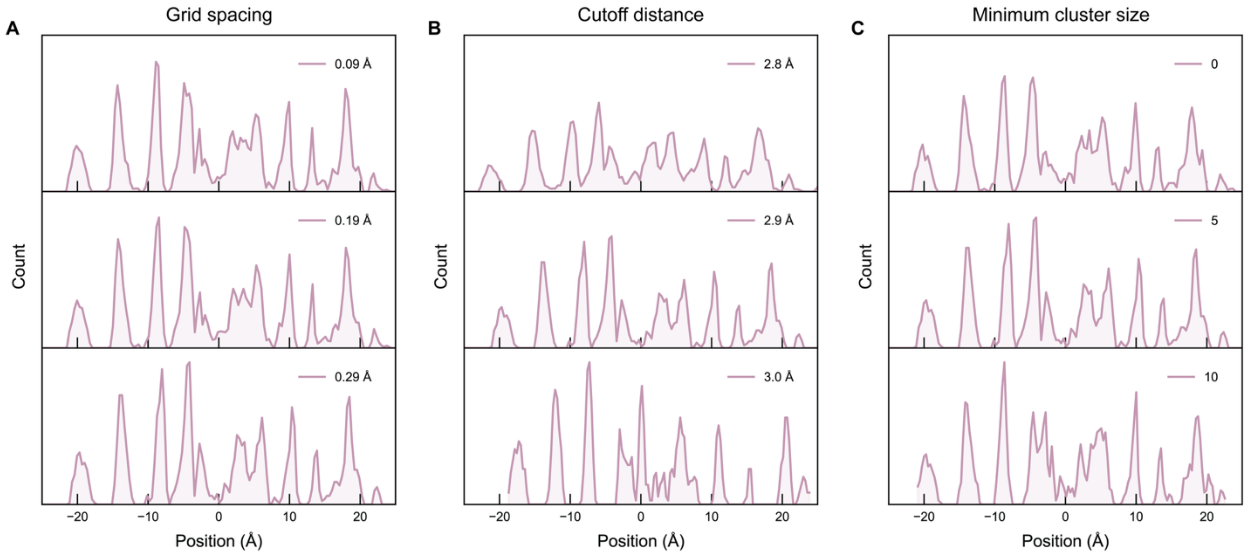

Figure S15: Effect of main algorithmic parameters on the void PC1 distribution. (**A**) Grid spacing: 0.09 Å, 0.19 Å, 0.29 Å, with the cutoff distance and minimum cluster size fixed at 2.9 Å and 5, respectively. (**B**) Cutoff distance: 2.8 Å, 2.9 Å, 3.0 Å, with the grid spacing and minimum cluster size fixed at 0.19 Å and 5, respectively. (**C**) Minimum cluster size: 0, 5, 10, with the grid spacing and cutoff distance fixed at 0.19 Å and 2.9 Å, respectively. These parameters introduce some variation in the detailed shape of the distributions; however, changes in grid spacing and minimum cluster size do not shift the positions of the main peaks. In contrast, the cutoff distance has a larger effect on peak positions because it determines which neighboring void sites are merged into the same cluster, thereby altering the spatial centres of the resulting void domains. Despite these differences, the void spatial pattern remain consistent across all tested parameter sets.

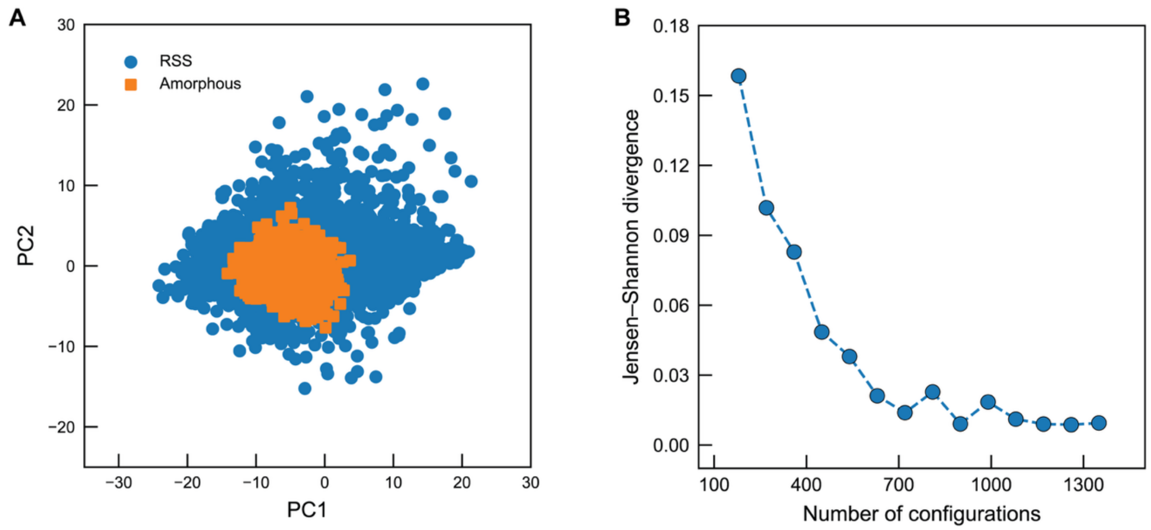

Figure S16: Analysis of structural diversity and sampling convergence in the RSS dataset. (**A**) PCA of atom-wise SOAP descriptors for RSS (blue) and amorphous (orange) configurations. Each point represents a local atomic environment. (**B**) Jensen–Shannon divergence as a function of the number of sampled RSS configurations, showing convergence of the explored structural distribution.

Supplementary Table

Table S1: Comparison of lattice constants and relative errors for the gray and black allotropes of As, computed using various DFT functionals. All relative errors are computed with respect to experimental values. In the case of r^2SCAN, we note a strong overestimation of the *c* lattice parameter of gray As (marked by * in the table), which appears to reflect a missing description of the van der Waals attraction between the layers. For gray As, we tested the effect of different *k*-point mesh densities, ranging from 4 × 4 × 2 to 10 × 10 × 3 and 20 × 20 × 6, corresponding to *k*-point spacing of 0.5 Å^{-1}, 0.2 Å^{-1}, and 0.1 Å^{-1}, respectively. Interestingly, the coarsest grid provides the closest prediction of the *c*-axis, with a relative error of 1.3%, whereas the finer grids (0.2 Å^{-1} and 0.1 Å^{-1}) yield similar results with much larger deviations (~45%), suggesting that numerical noise in r^2SCAN may be smoothed out by a coarser mesh.

	Methods	Lattice parameters (Å)			Relative errors (%)		
		a	*b*	*c*	*a*	*b*	*c*
Gray As	Experiment[S18]	3.760	3.760	10.547	—	—	—
	r^2SCAN	3.588	3.588	15.338*	–4.6	–4.6	45.4*
	r^2SCAN+rVV10	3.772	3.772	10.603	0.3	0.3	0.5
	TPSS	3.803	3.803	10.635	1.1	1.1	0.8
	PBE	3.826	3.826	10.645	1.8	1.8	0.9
	PBE-D3	3.794	3.794	10.058	0.9	0.9	–4.6
Black As	Experiment[S19]	3.620	10.850	4.480	—	—	—
	r^2SCAN	3.650	11.387	4.740	0.8	4.9	5.8
	r^2SCAN+rVV10	3.662	11.154	4.622	1.2	2.8	3.2
	TPSS	3.727	11.050	4.473	3.0	1.8	–0.2
	PBE	3.712	11.491	4.657	2.5	5.9	4.0
	PBE-D3	3.746	10.841	4.289	3.5	–0.1	–4.2

Supplementary References

S1. Dronskowski, R.; Bloechl, P. E. Crystal orbital Hamilton populations (COHP): energy-resolved visualization of chemical bonding in solids based on density-functional calculations. *J. Phys. Chem.* **1993**, *97*, 8617–8624.

S2. Müller, P. C.; Ertural, C.; Hempelmann, J.; Dronskowski, R. Crystal Orbital Bond Index: Covalent Bond Orders in Solids. *J. Phys. Chem. C* **2021**, *125*, 7959–7970.

S3. Maintz, S.; Deringer, V. L.; Tchougréeff, A. L.; Dronskowski, R. LOBSTER: A tool to extract chemical bonding from plane-wave based DFT. *J. Comput. Chem.* **2016**, *37*, 1030–1035.

S4. Nelson, R.; Ertural, C.; George, J.; Deringer, V. L.; Hautier, G.; Dronskowski, R. LOBSTER: Local orbital projections, atomic charges, and chemical-bonding analysis from projector-augmented-wave-based density-functional theory. *J. Comput. Chem.* **2020**, *41*, 1931–1940.

S5. George, J.; Petretto, G.; Naik, A.; Esters, M.; Jackson, A. J.; Nelson, R.; Dronskowski, R.; Rignanese, G.-M.; Hautier, G. Automated Bonding Analysis with Crystal Orbital Hamilton Populations. *ChemPlusChem* **2022**, *87*, e202200123.

S6. Naik, A. A.; Ueltzen, K.; Ertural, C.; Jackson, A. J.; George, J. LobsterPy: A package to automatically analyze LOBSTER runs. *J. Open Source Softw.* **2024**, *9*, 6286.

S7. Wiberg, N. *Lehrbuch der Anorganischen Chemie*; De Gruyter: Berlin, Boston, 2008.

S8. Osters, O.; Nilges, T.; Bachhuber, F.; Pielnhofer, F.; Weihrich, R.; Schöneich, M.; Schmidt, P. Synthesis and Identification of Metastable Compounds: Black Arsenic—Science or Fiction? *Angew. Chem. Int. Ed.* **2012**, *51*, 2994–2997.

S9. Zhou, Y.; Elliott, S. R.; Deringer, V. L. Structure and Bonding in Amorphous Red Phosphorus. *Angew. Chem. Int. Ed.* **2023**, *62*, e202216658.

S10. Ning, J.; Kothakonda, M.; Furness, J. W.; Kaplan, A. D.; Ehlert, S.; Brandenburg, J. G.; Perdew, J. P.; Sun, J. Workhorse minimally empirical dispersion-corrected density functional with tests for weakly bound systems: r^2SCAN+rVV10. *Phys. Rev. B* **2022**, *106*, 075422.

S11. Horton, M. K.; Huck, P.; Yang, R. X.; Munro, J. M.; Dwaraknath, S.; Ganose, A. M.; Kingsbury, R. S.; Wen, M.; Shen, J. X.; Mathis, T. S.; et al. Accelerated data-driven materials science with the Materials Project. *Nat. Mater.* **2025**, *24*, 1522–1532.

S12. Yoshiasa, A.; Tokuda, M.; Misawa, M.; Shimojo, F.; Momma, K.; Miyawaki, R.; Matsubara, S.; Nakatsuka, A.; Sugiyama, K. Natural arsenic with a unique order structure: potential for new quantum materials. *Sci. Rep.* **2019**, *9*, 6275.

S13. Wang, C.; Bai, S.; Zhao, C.; Yu, W.; Yang, Y.; Chen, Y.; Niu, C.-Y. Arsenic K4 crystal: A new stable direct-gap semiconductor allotrope. *Solid State Commun.* **2021**, *323*, 114128.

S14. Smith, P. M.; Leadbetter, A. J.; Apling, A. J. The structures of orthorhombic and vitreous arsenic. *Philos. Mag.* **1975**, *31*, 57–64.

S15. Bellissent, R.; Tourand, G. Étude de l'ordre local dans l'arsenic amorphe par diffraction de neutrons. *J. Phys. France* **1976**, *37*, 1423–1426.

S16. Batatia, I.; Benner, P.; Chiang, Y.; Elena, A. M.; Kovács, D. P.; Riebesell, J.; Advincula, X. R.; Asta, M.; Avaylon, M.; Baldwin, W. J.; et al. A foundation model for atomistic materials chemistry. *J. Chem. Phys.* **2025**, *163*, 184110.

S17. Krebs, H.; Steffen, R. Neubestimmung der Nahordnung im glasigen Selen, im explosiven Antimon und im β- und γ-Arsen. *Z. Anorg. Allg. Chem.* **1964**, *327*, 224–237.

S18. Schiferl, D.; Barrett, C. S. The crystal structure of arsenic at 4.2, 78 and 299°K. *J. Appl. Crystallogr.* **1969**, *2*, 30–36.

S19. Krebs, H.; Holz, W.; Worms, K. H. Über die Struktur und die Eigenschaften der Halbmetalle, X. Eine neue rhombische Arsenmodifikation und ihre Mischkristallbildung mit schwarzem Phosphor. *Chem. Ber.* **1957**, *90*, 1031–1037.
